# Supplementary material for: Understanding patterns of fatigue in health and disease: protocol for an ecological momentary assessment study using digital technologies
Source: BMJ Open. 2024 May 27;14(5):e081416. doi: 10.1136/bmjopen-2023-081416 (PMC11131111; doi:10.1136/bmjopen-2023-081416)
Supplement: Supplementary data [file bmjopen-2023-081416supp001.pdf]

UNIVERSITY OF ABERDEEN

Participant identification number

# RECORD OF PARTICIPANT'S VERBAL CONSENT

## Understanding Patterns of Fatigue in Health and Disease

The following is a telephone script and record of verbal consent for participants who indicate to the researchers that they would like to participate in the study. A copy of this consent form will be scanned or photocopied and sent to the participant via email or by post (according to their preference). Researcher introduces themself and the study and checks whether it is an appropriate time to conduct the consent process. Researcher checks participant's name and asks permission to audio-record the consent process. Audio-recorder is then turned on.

Chief Investigator: Dr Rosalind Adam

Researcher initials

1. I confirm that I have read and understand the information sheet (version .......... date ...............) for the above study, have had the opportunity to ask questions, and have had these answered satisfactorily. ☐
2. I understand that my participation is voluntary and that I am free to withdraw at any time, without giving any reason, without my medical care or legal rights being affected. Only anonymised data collected up until the point of withdrawal may still be used in analysis. ☐
3. I agree to provide data collected via a wearable patch and a wrist worn device ☐
4. I agree to provide ratings of my fatigue via a smartphone app ☐
5. I agree to taking part in an interview with a researcher at the end of the study ☐
6. I agree to my interview being audio-recorded. I understand that my recording will be kept confidential and that anonymous quotations from recordings may be used in presentations and publications. ☐
7. I agree to take part in a "feedback session" after my data have been analysed. I agree to this session being audio recorded. I understand that my recording will be kept confidential and that anonymous quotations from this session may be used in publications and presentations Yes ☐ No ☐

Verbal Consent, Version 2, 14th October 2022 IRAS Project ID: 317198

8. I agree that Information about me can be stored on University of Aberdeen computer servers. ☐

9. I agree that my data can be shared with the collaborating investigators from University College London, University of Cambridge, University of Lancaster, Southampton University, University of Glasgow and Panoramic Digital Health. I understand that identifiable information such as contact details will only be shared when it is necessary for a research team member from one of these organisations to contact me about the study (for example, in the feedback session). ☐

10. I agree that researchers can contact my general practitioner in the case that any incidental findings are noted during the study (for example, ECG changes that the researchers were not seeking to find or investigating, but that have been noted by researchers and which might be medically relevant) ☐

11. I understand that data collected during the study may be looked at by individuals from the University of Aberdeen, the regulatory authorities or from NHS, where it is relevant to my taking part in this research. I give permission for these individuals to have access to this data. ☐

11. I would like to be invited to take part in future ethically reviewed and approved research related to this study. I understand identifiable contact information will be kept after the end of this study and this information will be held in accordance with data protection regulation. Yes ☐ No ☐

12. I agree to take part in the above study ☐

(If Participant agrees to participate, researcher asks the participant to confirm their full name and signs and dates below)

Name of Participant ______________________________________________________

| | | |
|---|---|---|
| Name of Researcher | Signature of Researcher | Date and Time |

Verbal Consent, Version 2, 14th October 2022 IRAS Project ID: 317198

Verbal Consent, Version 2, 14th October 2022 IRAS Project ID: 317198
