# Supplementary material for: Understanding patterns of fatigue in health and disease: protocol for an ecological momentary assessment study using digital technologies
Source: BMJ Open. 2024 May 27;14(5):e081416. doi: 10.1136/bmjopen-2023-081416 (PMC11131111; doi:10.1136/bmjopen-2023-081416)
Supplement: Supplementary data [file bmjopen-2023-081416supp002.pdf]

Supplementary data file 2: Selecting technologies for the study

Selection of the technologies and platforms considered as part of the procurement process

- Philips health band https://www.usa.philips.com/healthcare/product/HC422210064081/philips-health-band#specifications
- Philips Actiwatch 2
- M-Path https://m-path.io/landing/
- PRO Diary CamNtech PRO-Diary - CamNtech
- LifeData experience platform LifeData Experience Sampling App - LifeData Experience Sampling App for Research (lifedatacorp.com)
- MediBiosense ECG patch technology:
- Movisens EcgMove4, Move 4, SensorTrigger, EdaMove 4, LKightMove4 Products - movisens GmbH

Key factors that were considered when choosing technologies to capture project data

- Range of data parameters that can be captured or measured
- Study team experience and knowledge of the technology and successful deployment in other projects
- Cost
- Support infrastructure for technical problems
- Ease of data download/supports data flow within the trial, maintaining privacy and security
- GDPR compliance
- Accuracy (e.g. consensus that wrist-worn heart rate monitoring lacked the accuracy required for our study)
- Ease of use/setting up for end user/requirement for participant internet connectivity for successful deployment
- Ability to interact with raw data from devices/apply and test different algorithms to raw data
