# Supplementary material for: Understanding patterns of fatigue in health and disease: protocol for an ecological momentary assessment study using digital technologies
Source: BMJ Open. 2024 May 27;14(5):e081416. doi: 10.1136/bmjopen-2023-081416 (PMC11131111; doi:10.1136/bmjopen-2023-081416)
Supplement: Supplementary data [file bmjopen-2023-081416supp003.pdf]

TOPIC GUIDE FOR END OF STUDY INTERVIEWS

Understanding Patterns of Fatigue in Health and Disease

Interviews will be directed to some extent by the participants. This document sets out topics that the interviewer will aim to cover. It will be used flexibly.

INTRODUCTIONS

- Introductions and reminder that interview can be paused or stopped at any time. Audio-recording device activated.
- In this interview, we would like to ask you about your experiences of fatigue over the study period and your opinions about taking part in the study and using the study devices.

GENERAL CONTEXT

- How have you been in general over the past two weeks?
- Have these been typical weeks for you (prompts might include usual activity levels/work/typical levels of fatigue)

FATIGUE EXPERIENCE

- Tell me about (any) fatigue that you have experienced over the last two weeks? Prompts might include: severity, how often fatigue was experienced, whether fatigue was mainly mental/physical and how it felt in the body.

If fatigue was experienced (control group participants may not have experienced fatigue):

- Do you remember any specific day to have been particularly more difficult or particularly good (in terms of fatigue)?
- Can you think of any triggers or things that might have worsened your fatigue? (prompts might include: physical activity, sleep, medications, stress, treatment cycle, menstruation, weather)
- How did the fatigue impact your physical activity?
- How did the fatigue impact your cognitive ability?
- What did you do to cope with your fatigue or any things that have made the fatigue better/helped?

FEASIBLITY

We would like to will discuss more specifically about some of the devices and ways that we have collected your information throughout the study.

- Did you face any difficulties using any of the devices? (Prompted to describe these, prompts might include app set up, ECG patch set up, use of app, whether the devices were uncomfortable or hindered activities/movements).

1. Panoramic Digital Health Bracelet and Bluetooth Tags
   - What did you like most about using the bracelet?
   - What did you not like about the device?
   - How could it be improved?
   - How easy or difficult it was to set up and use?

2. ECG Patch
   - What did you like about using the ECG Patch?
   - What did you not like, and could be different?
   - How easy or difficult was it to use?

3. Daily fatigue scores (m-Path)
   - Tell me about your experiences of using the app to enter fatigue scores. Prompts might include:
     - How easy/difficult it was to use the app
     - How easy/difficult was it to enter the fatigue scores? Why?
     - Whether the number and timings of the ratings were convenient/inconvenient, whether the ratings were too frequent/not frequent enough/just right to capture the difference/variability in fatigue
     - Whether the questions were easy to understand
     - Can you think of any other questions that could better capture your daily fatigue? What and why?
     - Is there anything that you did you not like about scoring our fatigue daily? What could be done better?
     - How convenient was it to use the app?
     - Would you rather have preferred a different method to input your daily scores? How? Why?

4. We used three questionnaires at the start and end of the study (participant is reminded about the nature of the questionnaires). Did you find the questions in these questionnaires easy to understand? Do you think the questions were relevant to you?

SUMMING UP

- Overall, how have you found participating in this study?
  - Any positive things about taking part
  - Any negative things about taking part
- We have covered lots of things (give brief summary); is there anything else that you would like to speak about that we haven't covered?

Participant is thanked for their time and reminded that they will have the opportunity to get a feedback session with a senior researcher to go over the data they have provided.

END of INTERVIEW
