# Supplementary material for: Understanding patterns of fatigue in health and disease: protocol for an ecological momentary assessment study using digital technologies
Source: BMJ Open. 2024 May 27;14(5):e081416. doi: 10.1136/bmjopen-2023-081416 (PMC11131111; doi:10.1136/bmjopen-2023-081416)
Supplement: Author's manuscript [file bmjopen-2023-081416.draft_revisions.pdf]

BMJ Open is committed to open peer review. As part of this commitment we make the peer review history of every article we publish publicly available.

When an article is published we post the peer reviewers' comments and the authors' responses online. We also post the versions of the paper that were used during peer review. These are the versions that the peer review comments apply to.

The versions of the paper that follow are the versions that were submitted during the peer review process. They are not the versions of record or the final published versions. They should not be cited or distributed as the published version of this manuscript.

BMJ Open is an open access journal and the full, final, typeset and author-corrected version of record of the manuscript is available on our site with no access controls, subscription charges or pay-per-view fees (http://bmjopen.bmj.com).

If you have any questions on BMJ Open's open peer review process please email info.bmjopen@bmj.com

BMJ Open

Understanding patterns of fatigue in health and disease: Protocol for an Ecological Momentary Assessment Study using digital technologies

Journal:	*BMJ Open*
Manuscript ID	bmjopen-2023-081416
Article Type:	Protocol
Date Submitted by the Author:	23-Nov-2023
Complete List of Authors:	Adam, Rosalind; University of Aberdeen College of Life Sciences and Medicine, Academic Primary Care<br>Lotankar, Yojana; University of Westminster, Psychology and Neuroscience<br>Sas, Corina; Lancaster University<br>Powell, Daniel; University of Aberdeen<br>Martinez, Veronica; University of Cambridge, Department of Engineering<br>Green, Stephen; University of Cambridge, Department of Engineering<br>Cooper, Jonathan; University of Glasgow<br>Bradbury, Katherine; University of Southampton, Psychology<br>Sive, Jonathan; University College London Hospitals NHS Foundation Trust<br>Hill, Derek; University College London Faculty of Engineering Sciences, Medical Physics & Biomedical engineering; Panoramic Digital Health Ltd.
Keywords:	Fatigue, Information technology < BIOTECHNOLOGY & BIOINFORMATICS, Heart failure < CARDIOLOGY, Myeloma < HAEMATOLOGY, Patient Reported Outcome Measures, Post-Acute COVID-19 Syndrome

SCHOLARONE™
Manuscripts

Understanding patterns of fatigue in health and disease: Protocol for an Ecological Momentary Assessment Study using digital technologies

Rosalind Adam, Institute of Applied Health Sciences, University of Aberdeen, United Kingdom

Yojana Lotankar, Psychology and Neuroscience, University of Westminster, London, United Kingdom

Corina Sas, School of Computing and Communications, Lancaster University, United Kingdom

Daniel Powell, Institute of Applied Health Sciences, University of Aberdeen, United Kingdom

Veronica Martinez, Department of Engineering, University of Cambridge, United Kingdom

Stephen Green, Department of Engineering, University of Cambridge, United Kingdom

Jonathan Cooper, James Watt School of Engineering, University of Glasgow, United Kingdom

Katherine J Bradbury, Psychology, University of Southampton, United Kingdom

Jonathan Sive, Consultant Haematologist, University College Hospitals, London, United Kingdom

Derek Hill, Medical Physics & Biomedical engineering, University College London, United Kingdom and Panoramic Digital Health, Grenoble, France

Word Count - 2894

For peer review only

For peer review only - http://bmjopen.bmj.com/site/about/guidelines.xhtml

Abstract

Introduction

Fatigue is prevalent across a wide range of medical conditions and can be debilitating and distressing. It is likely that fatigue is experienced differently according to the underlying aetiology, but this is poorly understood. Digital health technologies present a promising approach to give new insights into fatigue.

The aim of this study is to use digital health technologies, real-time self-reports, and qualitative interview data to investigate how fatigue is experienced over time in participants with myeloma, long COVID, heart failure, and in controls without problematic fatigue. Objectives are to understand which sensed parameters add value to the characterisation of fatigue and to determine whether study processes are feasible, acceptable, and scalable.

Methods and Analysis

An ecological momentary assessment study will be carried out over two or four weeks (participant defined). Individuals with fatigue relating to myeloma (n=10), heart failure (n=10), long COVID (n=10) and controls without problematic fatigue or a study condition (n=10) will be recruited. ECG patches will measure heart rate variability, respiratory rate, body temperature, activity, and posture. A wearable bracelet accompanied by environment beacons will measure physical activity, sleep, and room location within the home. Self-reports of mental and physical fatigue will be collected via smartphone app four times daily and on-demand. Validated fatigue and affect questionnaires will be completed at baseline and two weeks. End of study interviews will investigate experiences of fatigue and study participation. A feedback session will be offered to participants to discuss their data.

Data will be analysed using multilevel modelling and Machine Learning. Interviews and feedback sessions will be analysed using content or thematic analyses.

For peer review only - http://bmjopen.bmj.com/site/about/guidelines.xhtml

Ethics and dissemination: Approved by East of England – Cambridge East Research Ethics Committee (22/EE/0261). Results will be disseminated in peer reviewed journals and at international conferences.

Registration: Clinicaltrials.gov NCT05622669

Strengths and Limitations of this Study

- Integrates granular data from digital sensing technologies with participant self-reports and in-depth qualitative data
- Will use artificial intelligence techniques alongside multilevel modelling to detect patterns within temporal data
- Allows participants to reflect on their sensed data during bespoke feedback sessions, using qualitative data from these sessions to inform data interpretation
- Is not powered to quantify significant differences in fatigue ratings between groups of participants with different conditions

For peer review only

For peer review only - http://bmjopen.bmj.com/site/about/guidelines.xhtml

Introduction

Fatigue is a major clinical problem and is prevalent in most chronic medical conditions. Problematic fatigue is experienced by up to 98% of individuals with myeloma (1), around 50% of those with heart failure (2) and over 70% of those with long COVID (3). Fatigue is a multifaceted, fluctuating symptom which embodies biological, biochemical, physiological, psychological, emotional, and behavioural processes (4). Fatigue is distressing and causes reduced quality of life (5), and diminished economic productivity (6).

Fatigue is considered as a single, poorly defined symptom, but fatigue as a symptom is likely to encapsulate multidimensional experiences and fatigue is unlikely to be a single entity. There are likely to be specific patterns or characteristics of fatigue that vary according to the underlying mechanisms. For example, Powell et al. showed that individuals with multiple sclerosis (MS) were more likely to have fatigue that peaked in the afternoon, that came on more suddenly, and that was more likely to be present after physical activity compared to healthy controls (7).

People with long COVID commonly describe triggers that worsen fatigue, such as physical activity, stress, and sleep disturbance (8,9). In people with heart failure, tissue hypoperfusion and a mismatch between catabolic and anabolic processes can cause skeletal myopathy (10). This is thought to contribute to fatigue, particularly during physical exertion, and fatigue is often experienced alongside breathlessness (10). Fatigue is a common side effect of myeloma therapies, and during first treatment for myeloma, targeted medicines, chemotherapy agents, and steroids are often given in cycles (11). Fatigue is likely to vary during treatment cycles (12), but this is under-explored.

Despite its high prevalence, fatigue is under-researched and poorly understood (13). Fatigue research can be challenging because fatigue is changeable, subjective, and influenced by a wide range of individual, environmental, and external factors. Fatigue also poses clinical

For peer review only - http://bmjopen.bmj.com/site/about/guidelines.xhtml

challenges. When individuals present to clinicians with undifferentiated “tiredness”, standard diagnostic tests are usually unhelpful (14,15). There are limited personalised treatment options for fatigue and fatigue often remains untreated (16).

Ecological momentary assessment (EMA) studies collect relatively intensive, repeated, real-time data from participants who are living their everyday life (17). EMA methods have been used to gain insights into fatigue in several chronic medical conditions including renal failure(18) , MS (7)and cancer (19). EMA studies have the advantage of minimising participant recall bias and highlighting temporal variations within data (17). Few EMA studies have attempted to make comparisons between individuals with different clinical diagnoses. Furthermore, advances in digital health technologies offer new opportunities to combine participant self-reports of symptoms with real-time objective physiological and environmental data.

This paper describes the protocol for an EMA study of fatigue. Digital health technologies will be used to capture in-depth objective physiological, activity, self-report, and environmental measurements from individuals with myeloma, long COVID, heart failure, and a control group without these conditions. These medical conditions have been chosen because they are likely to involve different underlying mechanisms of fatigue, and different patterns of fatigue.

The aim of the study is to measure fatigue within individuals over time, to quantify variations in fatigue levels within individuals, and to describe relationships between patient-reported fatigue, patient-reported triggers for fatigue, and sensed parameters (including activity levels, sleep, heart rate variability, and other physiological parameters). Qualitative and quantitative data will be combined to investigate differences in the fatigue experience within and between individuals over time.

For peer review only - http://bmjopen.bmj.com/site/about/guidelines.xhtml

This study is exploratory and will test the feasibility, acceptability and practicalities of deploying the digital technologies and of the study procedures with a view to conducting a large-scale investigation into replicable, measurable, quantitative differences in fatigue patterns between groups of individuals with different medical conditions. The study will also develop artificial intelligence (AI) algorithms and explore the value of AI techniques in analysing and interpreting EMA study data.

Methods

Study design and setting

This EMA study will be conducted remotely, enrolling participants from anywhere within the United Kingdom (UK). Participants will self-rate their physical and cognitive fatigue four times daily and on demand over two or four weeks (according to participant preference). The study is an exploratory feasibility study, and the primary objective is to use both quantitative and qualitative methods to characterise lived experiences of fatigue and to explore temporal patterns in fatigue within individuals with different medical conditions.

Outcomes

The primary outcome is descriptive and concerns how patient reports of fatigue change over time, and the temporal relationships between fatigue, self-reported triggers for fatigue, and sensed parameters (see below).

Secondary outcomes relate to the feasibility of the study procedures. Secondary outcomes include ability to recruit eligible participants at the required rate; attrition rate; suitability of the study questionnaires and patient reported outcome measures (PROMS); acceptability and desirability of the study procedures; usability of the technologies; logistics of remote study assessments and data collection; and potential predictors of missing self-report data during the EMA study.

For peer review only - http://bmjopen.bmj.com/site/about/guidelines.xhtml

For peer review only

Sample Size

The study will aim to enrol 40 participants, including 10 participants with myeloma, 10 with heart failure, 10 with long COVID, and 10 control participants. It may be the case that the sample sizes in the groups are not evenly matched. This will be determined by participant interest and response. It is also possible that some participants provide limited data or have high levels of missing data (for example, due to sensor failure or poor adherence to EMA ratings). The research team may decide to recruit additional participants into a group in which participants have high levels of missing data, where missing data precludes meaningful insights into the participant's fatigue experience, and this will be judged during the study by the lead researcher.

This is a feasibility study without a quantitative primary outcome measure. As such, a formal sample size calculation is not required, however the sampling design has been chosen to sufficiently capture the expected within-day variability in fatigue following discussions with patient partners. In parallel group pilot trials, a sample size of 12 participants per group is suggested as a "rule of thumb" that will give adequate data to inform future definitive studies (20). The EMA study design employed here differs from traditional parallel group studies in that multiple observations are taken in the same individual over time, allowing for detailed within person descriptions of the phenomenon and temporal relationships/associations. In this study, granular patient reported outcome data will be combined with rich qualitative data. The estimate of ten patients per group is a pragmatic one that considers the primary study objective, researcher capacity, study duration, number of daily EMA ratings, and availability of sensors and other technical equipment.

Eligibility Criteria

Inclusion and exclusion criteria are listed in Table 1. Eligible participants are adults with fatigue (considered by them to be worse than "normal tiredness") related to myeloma, long

For peer review only - http://bmjopen.bmj.com/site/about/guidelines.xhtml

COVID, or heart failure, and control group participants without a study condition who do not experience problematic fatigue (worse than “normal tiredness”).

Table 1: Inclusion and Exclusion Criteria

Group allocation	Inclusion criteria	Exclusion criteria
All participants	Willing to participate in intermittent ecological momentary assessments of fatigue, to wear an electrocardiogram (ECG) patch, to provide questionnaire responses	Difficulty communicating in English<br>Adults lacking capacity to consent<br>Under 18 years of age<br>Declines to participate<br>Under investigation for or starting treatment for an endocrine, metabolic, or thyroid condition where the participant has not been established on a stable therapeutic dose of a licensed therapy for that condition<br>A confirmed diagnosis of sleep apnoea or narcolepsy<br>HADs depression and anxiety score at baseline greater than 8 on the depression questions (21), which might indicate untreated or undertreated depression<br>Shift work that involves overnight working between the hours of 9pm and 9am
Group A, individuals with myeloma	A confirmed diagnosis of myeloma<br>Has experienced fatigue that is perceived by the participant to be worse than “normal tiredness” and that they associate with myeloma or treatment for myeloma	Uncontrolled hypercalcaemia<br>Current or previous diagnosis of heart failure or long COVID<br>An active primary cancer diagnosis other than myeloma
Group B, Individuals with heart failure	A formal diagnosis of heart failure<br>All stages of heart failure and all aetiologies and with no specific ejection fraction cut-off<br>Has experienced fatigue that is perceived by the participant to be worse than “normal tiredness” and that they associate with their cardiac disease or its treatment	A current or previous diagnosis of myeloma or long COVID<br>Active cancer
Group C, Individuals with long COVID	Experiencing fatigue that is perceived by the participant to be worse than “normal tiredness”, with or without other physical or psychological symptoms that developed during or after an infection consistent with COVID-19<br>The fatigue (plus or minus any other symptoms) has continued for greater or equal to 12 weeks, and is not explained by an alternative diagnosis	A current or previous diagnosis of myeloma or heart failure<br>Active cancer
Group D, control group	Individuals aged 18 years or over without the disease conditions specified in Groups A to C	Presence of myeloma or another active cancer, heart failure, or long COVID<br>One or more chronic medical conditions which are unstable, poorly controlled, AND perceived by the individual to be causing fatigue

For peer review only - http://bmjopen.bmj.com/site/about/guidelines.xhtml

		Persistent or severe fatigue symptoms that are perceived by the individual to be worse than "normal tiredness"<br>Taking sedating medications to manage anxiety or insomnia including but not limited to benzodiazepines or "Z" drugs, zopiclone, zolpidem, and others in this British National Formulary Class

Recruitment and consent

Eligible patients with myeloma will be invited to participate by clinicians from the haematology clinic at University College London Hospital. Eligible patients with heart failure will be invited to participate by clinicians working in NHS Grampian's heart failure nursing service. Participants with heart failure will also be recruited from General Practices in NHS Grampian, facilitated by the NHS Research Scotland Primary Care (NRS Primary Care) network.

Participants with myeloma, heart failure, or long COVID who took part in previous focus groups as part of this project and who have consented to being contacted about future related research will be invited to participate by email. Relevant charity and patient advocacy organisations will also be invited to share study information leaflets with their members.

Control group participants will be recruited via staff mailing lists at collaborating academic institutions, patient involvement groups, and from community walking organisations.

The study may be advertised on X (formerly known as Twitter) if recruitment targets are not achieved via other routes.

Individuals identified by NHS clinicians will be handed an invitation letter, information sheet, reply slip and reply-paid envelope by their clinicians and asked to contact the study team if they are interested in participating. Those recruited by General Practices will be identified by searches of electronic medical records and sent invitation packs by post. Those invited by email will receive the participant information sheet and invitation letter as an attachment and will be asked to reply by email to the study team to indicate their interest.

For peer review only - http://bmjopen.bmj.com/site/about/guidelines.xhtml

Interested individuals will have an initial telephone or Teams video call, during which a researcher will explain the study and answer any questions. Eligibility screening will be conducted, and for eligible participants who wish to participate, audio-recorded verbal consent will be taken. A paper consent form will be initialled and signed by the researcher and a copy will be sent to the participant.

Participants will be offered a £50 voucher as a token of thanks.

<u>Participant timeline and Data Collection Methods</u>

The participant timeline is summarised in Figure 1.

Baseline Assessments

At study baseline, participants will complete the Mental and Physical State and Trait Energy and Fatigue Scales (STEF) parts one and three (22–24); Positive and Negative Affect Scales (PANAS) for "past few weeks" time frame (25) and the Modified Fatigue Impact Scale (MFIS)(26). These questionnaires were chosen as they have been validated and administered together, they will give insights into both physical and mental fatigue, "usual" fatigue (as a complement to the EMA measures), emotions, and the impact of fatigue on the individual.

Sensors

Participants will be posted a study kit containing an android smartphone with data SIM, an ECG patch (Vital Patch, Vital Connect Inc, California, USA), a wearable bracelet containing accelerometer accompanied by four environment beacons (Panoramic Digital Health SAS, Grenoble France).

.The VitalPatch ECG is an approved medical device and will be worn for the first seven days of the study. The patch measures respiratory rate, a single lead ECG with RR interval, heart rate parameters (including heart rate variability), body temperature, activity levels (actimetry), and body position. The ECG patch communicates by Bluetooth with a dedicated app (Medibiosense Ltd, UK) pre-installed on the study phone.

For peer review only - http://bmjopen.bmj.com/site/about/guidelines.xhtml

The Panoramic digital health bracelet (Panoramic Digital Health SAS, Grenoble, France) is a wrist worn device containing a three-axis accelerometer, three axis magnetometer, three gyroscope, temperature sensor and atmospheric pressure sensor to measure physical activity and sleep. The bracelet is used alongside four Bluetooth beacons, which are placed within labelled positions within the participants' homes (e.g., kitchen, living room, bedroom, top of stairs). The beacons communicate with the bracelet and generate data about the wearer's proximity to each beacon, and also record temperature, sound level and light level in their location, providing environmental context to the bracelet sensor data.

EMA Assessments

Participants will use the m-path app (https://m-path.io), which is a GDPR compliant research tool developed by the Katholieke Universiteit Leuven. The app will be pre-loaded onto the study smartphone, or participants can opt to download this on their own device if they prefer. The app will be used to send participants short questionnaires four times daily to rate their cognitive fatigue and physical fatigue from zero to ten, and to note any associated triggers for improvements in or deteriorations in fatigue. The anchoring and wording of the self-rating questions is based on "state" fatigue items from the Mental and Physical State and Trait Energy and Fatigue Scale (22). A full summary of the self-rating questions is provided in text box 1.

M-path will send audible alerts to the smartphone at pre-determined intervals, spaced throughout the day and pre-agreed with the participant to fit with work or personal commitments. Participants will be able to miss self-reports and will be asked to add extra reports on-demand if they notice that their fatigue levels are better or worse than usual.

For peer review only - http://bmjopen.bmj.com/site/about/guidelines.xhtml

Text Box 1: Self-rating prompts within the m-Path app

1. Overall, how fatigued do you feel at the moment?
- I feel no fatigue, strongest feeling of fatigue ever (0-10 slider)

2. How do you feel right now with regard to your capacity to perform your typical PHYSICAL ACTIVITIES....
- I feel no fatigue – 0-10 slider – Strongest feelings of fatigue ever felt

3. How do you feel right now with regard to your capacity to perform your typical MENTAL ACTIVITIES....
- I feel no fatigue – 0-10 slider – Strongest feelings of fatigue ever felt

4. Is your fatigue better, the same, or worse than usual?

CONDITIONAL
- IF the same – no further questions
- IF better – question about factors making better
- IF worse – question about factors making worse

Factors making better:
Do you think that any of the following might have affected your current levels of fatigue (select all that apply)?
- Exercise or physical activity within the last day
- Exercise or physical activity more than a day ago
- Taking a medicine (please specify)
- Taking rest
- Taking caffeine
- Less emotions or stress
- My mood
- Spending time with other people
- The weather
- Quiet time
- Something else (please specify) – free text option

Factors making worse:
Do you think that any of the following might have affected your current levels of fatigue (select all that apply)?
- Exercise or physical activity within the last day
- Exercise or physical activity more than a day ago
- Taking a medicine (please specify)
- Emotions or stress
- My mood
- Tasks or work relating to my employment
- Tasks or work relating to my home life
- Spending time with other people
- The weather
- Noise
- Other (please specify)

End of study assessments

Participants complete all baseline questionnaires again after two weeks and participate in an end of study qualitative interview, conducted according to a schedule (Supplementary data

For peer review only - http://bmjopen.bmj.com/site/about/guidelines.xhtml

file 1). They are invited to participate in an optional feedback and “sense-making” session, during which a researcher provides them with feedback on the data provided and any interesting features of the data are discussed. Feedback sessions are audio-recorded and used in analyses.

Data Management and analysis

ECG patch data will be downloaded manually from the phone by the research team when the phone is returned. Raw encrypted data from the Panoramic bracelets are also downloaded directly from the bracelets by the research team, where data are decrypted, quality checked and analysed. Periods of non-wear are detected using the temperature sensor and accelerometer. The raw accelerometer data is processed using the open source OxWearables algorithms to measure step count per minute (27), sleep and level of activity (28). The beacon data is processed to label the processed bracelet sensor data with location in the home, by identifying the beacon with the highest Bluetooth received signal strength indicator (RSSI). Visual inspection of the output of these algorithms alongside the VitalPatch output can be viewed in a data annotator to identify features of interest in the data including timing of getting out of bed, timing of any stair climb, and timing of any period of immobility following movement in the home. m-Path app data are downloaded from an on-line study dashboard

Summary data can be generated from the Panoramic digital health platform, including: total sleep time, step count per hour over the day, time spent and activity in each room per day, time spent and activity when away from home. More granular data can also be determined including time taken to get out of bed, number of room transitions per day, average daily time climbing stairs (where applicable), cadence for walking, and total period of immobility following movements within the home, and whether the participant gets out of bed when they wake up at night (eg: to go to toilet or kitchen) or stay in bed. Data on directional change (e.g.

For peer review only - http://bmjopen.bmj.com/site/about/guidelines.xhtml

turning/changing walking direction), change in height (e.g.: going up stairs, walking up slopes) can also be generated

Patient feedback reports will be created manually, identifying potentially interesting or unexplained features from self-report and sensed data (for example, particularly high or low self-reported fatigue scores, or rapid changes in fatigue scores. Visualisations will be produced (format and content will be iterative and refined during the study based on participant feedback). Feedback sessions with DH, CS, or RA will be audio-recorded and used to give insights into the face validity of the sensed data and the fatigue experience more broadly.

Artificial intelligence (AI) algorithms will be created by VM and SG (University of Cambridge) to automatically handle multi-variety/multidimensional data, detect symptom fluctuation over time, and identify patterns, trends, and groups. New algorithms using machine learning (ML) will be developed and tested to understand patterns of fatigue and relationships between sensed parameters. Within and between person and within and between group differences will be analysed using multi-level modelling and machine learning.

Qualitative data from interview transcripts will be analysed using Framework and thematic analysis.

Multiple investigators will be involved in the analyses.

Patient and Public Involvement

Six on-line focus groups were conducted with individuals with fatigue (qualitative results will be reported separately). Participants with experience of a study condition gave insights into their lived experience of fatigue and their thoughts about using digital technologies to measure aspects of the fatigue experience. The results from the focus groups directly informed study design, including study duration. Some participants would find four weeks of

For peer review only - http://bmjopen.bmj.com/site/about/guidelines.xhtml

data collection onerous, whereas for others, two weeks of data collection would be insufficient to capture perceived patterns in their fatigue (e.g. during myeloma treatment cycles).

The focus groups also led to the inclusion of the participant feedback session. It was important to participants that their sensed and self-reported data were explained to them and that they were given the opportunity to reflect on this data with an experienced researcher.

A patient partner with myeloma liaised with the study team during the preparation of the study documents and gave feedback on the protocol and patient information sheet.

Ethics and dissemination

The study was approved by the East of England – Cambridge East Research Ethics Committee (22/EE/0261). The study team are interdisciplinary researchers spanning primary care (clinical), health psychology, engineering, medical physics, human computing interaction, and artificial intelligence. Results will be disseminated in a range of peer reviewed journals spanning these disciplines, and at international conferences.

Study Status

The study is currently recruiting. Participant recruitment commenced on 7th December 2022 and is expected to finish recruiting by 18th December 2023.

Funding Statement

This work is being funded by EPSRC. Grant reference EP/W003228/1. Dr Rosalind Adam is funded by a Chief Scientist Office (Scotland) Senior Clinical Academic Fellowship (reference SCAF/18/02)

Acknowledgements

The research team would like to thank all patient participants in our on-line focus groups for their input into the design of this study.

For peer review only - http://bmjopen.bmj.com/site/about/guidelines.xhtml

For peer review only

Competing Interests

Derek Hill, Professor of Digital Health at UCL, is also founder and CEO of Panoramic Digital Health, a company that is providing technology for this study. This potential conflict of interest is documented in accordance with UCL Disclosure of Conflict and Declaration of Interest Policy and has been reviewed by Sponsor.

None of the other collaborators have any conflicts to declare.

Author Contributions

RA wrote the initial draft. RA, KB, JC, DH, VM, and CS conceptualised the study. All authors reviewed the draft protocol, provided feedback and approved the final manuscript.

References

1. Ramsenthaler C, Kane P, Gao W, Siegert RJ, Edmonds PM, Schey SA, et al. Prevalence of symptoms in patients with multiple myeloma: a systematic review and meta-analysis. Eur J Haematol [Internet]. 2016 Nov 1 [cited 2022 Jul 15];97(5):416–29. Available from: https://pubmed.ncbi.nlm.nih.gov/27528496/
2. Barnes S, Gott M, Payne S, Parker C, Seamark D, Gariballa S, et al. Prevalence of symptoms in a community-based sample of heart failure patients. J Pain Symptom Manage [Internet]. 2006 Sep;32(3):208–16. Available from: http://www.ncbi.nlm.nih.gov/pubmed/16939845
3. Prevalence of ongoing symptoms following coronavirus (COVID-19) infection in the UK - Office for National Statistics [Internet]. [cited 2023 Feb 2]. Available from: https://www.ons.gov.uk/peoplepopulationandcommunity/healthandsocialcare/conditionsanddiseases/bulletins/prevalenceofongoingsymptomsfollowingcoronaviruscovid19infectionintheuk/2february2023
4. Swain MG. Fatigue in chronic disease. Clin Sci (Lond) [Internet]. 2000 Jul;99(1):1–8. Available from: http://www.ncbi.nlm.nih.gov/pubmed/10887052
5. Kluger BM, Krupp LB, Enoka RM. Fatigue and fatigability in neurologic illnesses: proposal for a unified taxonomy. Neurology [Internet]. 2013 Jan 22;80(4):409–16. Available from: http://www.ncbi.nlm.nih.gov/pubmed/23339207

For peer review only - http://bmjopen.bmj.com/site/about/guidelines.xhtml

6. Finsterer J, Mahjoub SZ. Fatigue in healthy and diseased individuals. Am J Hosp Palliat Care [Internet]. 2014 Aug;31(5):562–75. Available from: http://www.ncbi.nlm.nih.gov/pubmed/23892338

7. Powell DJH, Liossi C, Schlotz W, Moss-Morris R. Tracking daily fatigue fluctuations in multiple sclerosis: ecological momentary assessment provides unique insights. J Behav Med [Internet]. 2017 Oct;40(5):772–83. Available from: http://www.ncbi.nlm.nih.gov/pubmed/28281106

8. Ziauddeen N, Gurdasani D, O'Hara ME, Hastie C, Roderick P, Yao G, et al. Characteristics and impact of Long Covid: Findings from an online survey. PLoS One [Internet]. 2022 Mar 1 [cited 2023 Feb 2];17(3). Available from: https://pubmed.ncbi.nlm.nih.gov/35259179/

9. Sas C, Lotankar Y, Adam R, Bradbury KJ, Cooper J, Hill DL, et al. "A Switch Went off in my Whole Body": Lived Experiences of Fatigue and Post-Exertional Malaise in Long Covid. Extended Abstracts of the 2023 CHI Conference on Human Factors in Computing Systems [Internet]. 2023 Apr 19 [cited 2023 Apr 28];1–7. Available from: https://dl.acm.org/doi/10.1145/3544549.3585846

10. Clark AL. Origin of symptoms in chronic heart failure. Heart. 2006 Jan;92(1):12–6.

11. Goldman-Mazur S, Kumar SK. Current approaches to management of high-risk multiple myeloma. Am J Hematol [Internet]. 2021 Jul 1 [cited 2023 Feb 2];96(7):854–71. Available from: https://pubmed.ncbi.nlm.nih.gov/33725367/

12. Dombeck C, Swezey T, Gonzalez Sepulveda JM, Reeve BB, LeBlanc TW, Chandler D, et al. Patient perspectives on considerations, tradeoffs, and experiences with multiple myeloma treatment selection: a qualitative descriptive study. BMC Cancer [Internet]. 2023 Dec 1 [cited 2023 Feb 9];23(1). Available from: /pmc/articles/PMC9850680/

13. Matura LA, Malone S, Jaime-Lara R, Riegel B. A Systematic Review of Biological Mechanisms of Fatigue in Chronic Illness.

14. Kitai E, Blumberg G, Levy D, Golan-Cohen A, Vinker S. Fatigue as a first-time presenting symptom: management by family doctors and one year follow-up. Isr Med Assoc J [Internet]. 2012 Sep;14(9):555–9. Available from: http://www.ncbi.nlm.nih.gov/pubmed/23101419

15. Gialamas A, Beilby JJ, Pratt NL, Henning R, Marley JE, Roddick JF. Investigating tiredness in Australian general practice. Do pathology tests help in diagnosis? Aust Fam Physician [Internet]. 2003 Aug;32(8):663–6. Available from: http://www.ncbi.nlm.nih.gov/pubmed/12973880

16. Vaes AW, Goërtz YMJ, van Herck M, Beijers RJHCG, van Beers M, Burtin C, et al. Physical and mental fatigue in people with non-communicable chronic diseases. Ann Med [Internet]. 2022 [cited 2023 Apr 5];54(1):2522–34. Available from: https://pubmed.ncbi.nlm.nih.gov/36111684/

17. Shiffman S, Stone AA, Hufford MR. Ecological Momentary Assessment. Annu Rev Clin Psychol. 2008 Apr 1;4(1):1–32.

For peer review only - http://bmjopen.bmj.com/site/about/guidelines.xhtml

18. Abdel-Kader K, Jhamb M, Mandich LA, Yabes J, Keene RM, Beach S, et al. Ecological momentary assessment of fatigue, sleepiness, and exhaustion in ESKD. BMC Nephrol [Internet]. 2014 Feb 6 [cited 2023 Feb 3];15(1). Available from: https://pubmed.ncbi.nlm.nih.gov/24502751/

19. Kampshoff CS, Verdonck-de Leeuw IM, van Oijen MG, Sprangers MA, Buffart LM. Ecological momentary assessments among patients with cancer: A scoping review. Eur J Cancer Care (Engl). 2019 May;28(3):e13095.

20. Julious SA. Sample size of 12 per group rule of thumb for a pilot study. Pharm Stat [Internet]. 2005 Oct 1 [cited 2022 Jul 15];4(4):287–91. Available from: https://onlinelibrary.wiley.com/doi/full/10.1002/pst.185

21. Zigmond AS, Snaith RP. The Hospital Anxiety and Depression Scale. Acta Psychiatr Scand. 1983 Jun;67(6):361–70.

22. O'Connor P. Mental and physical state and trait energy scales. 2006.

23. Boolani A, Manierre M. An exploratory multivariate study examining correlates of trait mental and physical fatigue and energy. Fatigue. 2019 Jan 2;7(1):29–40.

24. Cook DB, O'Connor PJ, Lange G, Steffener J. Functional neuroimaging correlates of mental fatigue induced by cognition among chronic fatigue syndrome patients and controls. Neuroimage. 2007 May;36(1):108–22.

25. Watson D, Clark LA, Tellegen A. Development and validation of brief measures of positive and negative affect: The PANAS scales. J Pers Soc Psychol. 1988;54(6):1063–70.

26. Fisk JD, Ritvo PG, Ross L, Haase DA, Marrie TJ, Schlech WF. Measuring the Functional Impact of Fatigue: Initial Validation of the Fatigue Impact Scale. Clinical Infectious Diseases. 1994 Jan 1;18(Supplement_1):S79–83.

27. Small SR, Chan S, Walmsley R, Fritsch L von, Acquah A, Mertes G, et al. Development and Validation of a Machine Learning Wrist-worn Step Detection Algorithm with Deployment in the UK Biobank. medRxiv [Internet]. 2023 Jan 1;2023.02.20.23285750. Available from: http://medrxiv.org/content/early/2023/02/22/2023.02.20.23285750.abstract

28. Doherty A, Smith-Byrne K, Ferreira T, Holmes M V, Holmes C, Pulit SL, et al. GWAS identifies 14 loci for device-measured physical activity and sleep duration. Nat Commun. 2018 Dec 10;9(1):5257.

For peer review only

Figure 1: Participant timeline and flow chart

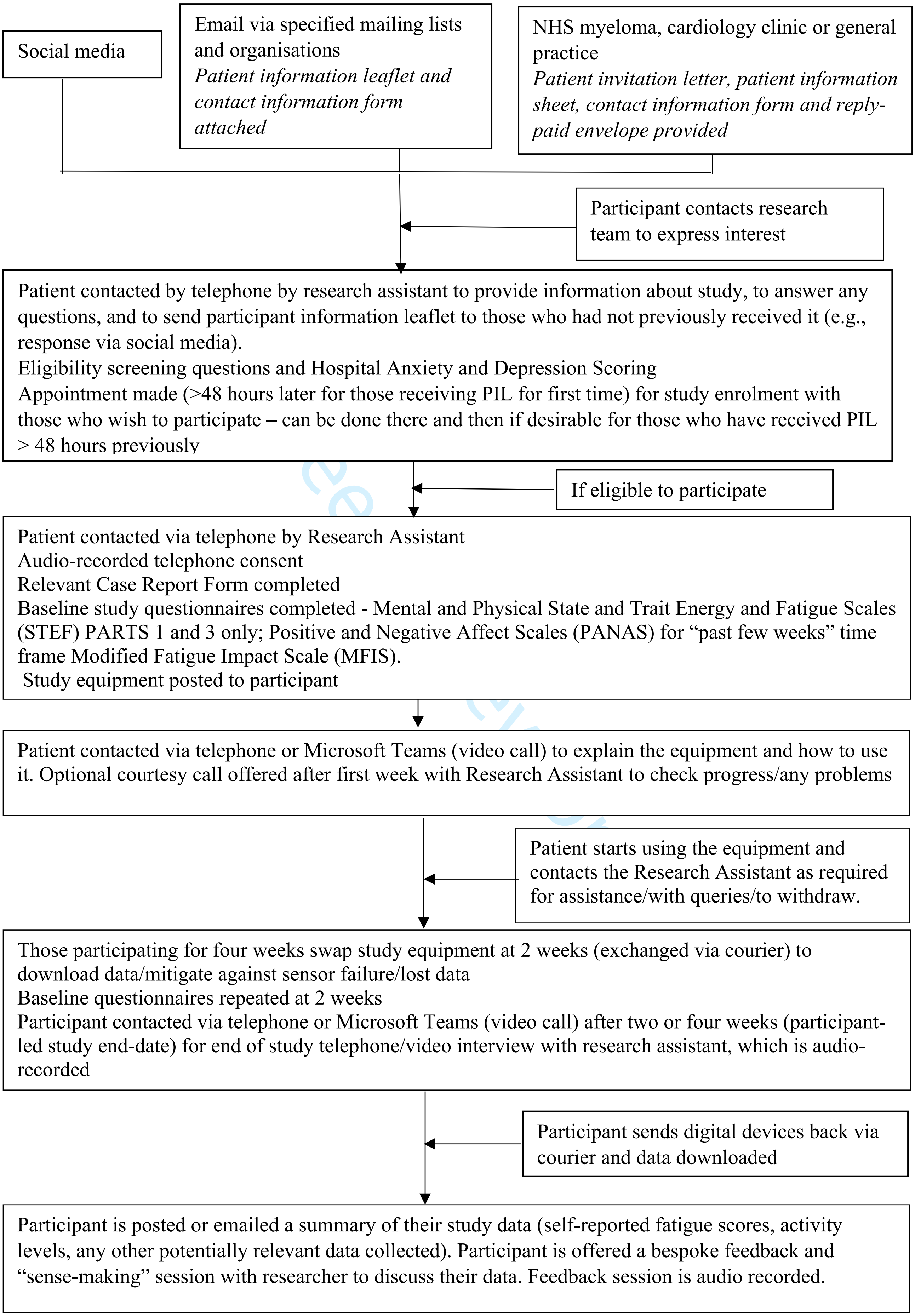

<u>**TOPIC GUIDE FOR END OF STUDY INTERVIEWS**</u>

UNIVERSITY OF ABERDEEN

Understanding Patterns of Fatigue in Health and Disease

Interviews will be directed to some extent by the participants. This document sets out topics that the interviewer will aim to cover. It will be used flexibly.

For peer review only

INTRODUCTIONS

- Introductions and reminder that interview can be paused or stopped at any time. Audio-recording device activated.
- In this interview, we would like to ask you about your experiences of fatigue over the study period and your opinions about taking part in the study and using the study devices.

GENERAL CONTEXT

- How have you been in general over the past two weeks?
- Have these been typical weeks for you (prompts might include usual activity levels/work/typical levels of fatigue)

FATIGUE EXPERIENCE

- Tell me about (any) fatigue that you have experienced over the last two weeks? Prompts might include: severity, how often fatigue was experienced, whether fatigue was mainly mental/physical and how it felt in the body.

If fatigue was experienced (control group participants may not have experienced fatigue):

- Do you remember any specific day to have been particularly more difficult or particularly good (in terms of fatigue)?
- Can you think of any triggers or things that might have worsened your fatigue? (prompts might include: physical activity, sleep, medications, stress, treatment cycle, menstruation, weather)
- How did the fatigue impact your physical activity?
- How did the fatigue impact your cognitive ability?
- What did you do to cope with your fatigue or any things that have made the fatigue better/helped?

FEASIBLITY

We would like to will discuss more specifically about some of the devices and ways that we have collected your information throughout the study.

- Did you face any difficulties using any of the devices? (Prompted to describe these, prompts might include app set up, ECG patch set up, use of app, whether the devices were uncomfortable or hindered activities/movements).

1. Panoramic Digital Health Bracelet and Bluetooth Tags
   - What did you like most about using the bracelet?
   - What did you not like about the device?
   - How could it be improved?
   - How easy or difficult it was to set up and use?

2. ECG Patch
   - What did you like about using the ECG Patch?
   - What did you not like, and could be different?
   - How easy or difficult was it to use?

3. Daily fatigue scores (m-Path)
   - Tell me about your experiences of using the app to enter fatigue scores. Prompts might include:
     - How easy/difficult it was to use the app
     - How easy/difficult was it to enter the fatigue scores? Why?
     - Whether the number and timings of the ratings were convenient/inconvenient, whether the ratings were too frequent/not frequent enough/just right to capture the difference/variability in fatigue
     - Whether the questions were easy to understand
     - Can you think of any other questions that could better capture your daily fatigue? What and why?
     - Is there anything that you did you not like about scoring our fatigue daily? What could be done better?
     - How convenient was it to use the app?
     - Would you rather have preferred a different method to input your daily scores? How? Why?

4. We used three questionnaires at the start and end of the study (participant is reminded about the nature of the questionnaires). Did you find the questions in these questionnaires easy to understand? Do you think the questions were relevant to you?

SUMMING UP

- Overall, how have you found participating in this study?
  - Any positive things about taking part
  - Any negative things about taking part
- We have covered lots of things (give brief summary); is there anything else that you would like to speak about that we haven't covered?

Participant is thanked for their time and reminded that they will have the opportunity to get a feedback session with a senior researcher to go over the data they have provided.

END of INTERVIEW

For peer review only

BMJ Open

Understanding patterns of fatigue in health and disease: Protocol for an Ecological Momentary Assessment Study using digital technologies

Journal:	*BMJ Open*
Manuscript ID	bmjopen-2023-081416.R1
Article Type:	Protocol
Date Submitted by the Author:	24-Apr-2024
Complete List of Authors:	Adam, Rosalind; University of Aberdeen College of Life Sciences and Medicine, Academic Primary Care<br>Lotankar, Yojana; University of Westminster, Psychology and Neuroscience<br>Sas, Corina; Lancaster University<br>Powell, Daniel; University of Aberdeen<br>Martinez, Veronica; University of Cambridge, Department of Engineering<br>Green, Stephen; University of Cambridge, Department of Engineering<br>Cooper, Jonathan; University of Glasgow<br>Bradbury, Katherine; University of Southampton, Psychology<br>Sive, Jonathan; University College London Hospitals NHS Foundation Trust<br>Hill, Derek; University College London Faculty of Engineering Sciences, Medical Physics & Biomedical engineering; Panoramic Digital Health Ltd.
**Primary Subject Heading**:	Health services research
Secondary Subject Heading:	Oncology, Cardiovascular medicine, Infectious diseases
Keywords:	Fatigue, Information technology < BIOTECHNOLOGY & BIOINFORMATICS, Heart failure < CARDIOLOGY, Myeloma < HAEMATOLOGY, Patient Reported Outcome Measures, Post-Acute COVID-19 Syndrome

SCHOLARONE™
Manuscripts

Understanding patterns of fatigue in health and disease: Protocol for an Ecological Momentary Assessment Study using digital technologies

Rosalind Adam, Institute of Applied Health Sciences, University of Aberdeen, United Kingdom. Corresponding author. Rosalindadam@abdn.ac.uk

Yojana Lotankar, Psychology and Neuroscience, University of Westminster, London, United Kingdom

Corina Sas, School of Computing and Communications, Lancaster University, United Kingdom

Daniel Powell, Institute of Applied Health Sciences, University of Aberdeen, United Kingdom

Veronica Martinez, Department of Engineering, University of Cambridge, United Kingdom

Stephen Green, Department of Engineering, University of Cambridge, United Kingdom

Jonathan Cooper, James Watt School of Engineering, University of Glasgow, United Kingdom

Katherine J Bradbury, Psychology, University of Southampton, United Kingdom

Jonathan Sive, Consultant Haematologist, University College Hospitals, London, United Kingdom

Derek Hill, Medical Physics & Biomedical engineering, University College London, United Kingdom and Panoramic Digital Health, Grenoble, France

Word Count - 3793

For peer review only

Abstract

Introduction

Fatigue is prevalent across a wide range of medical conditions and can be debilitating and distressing. It is likely that fatigue is experienced differently according to the underlying aetiology, but this is poorly understood. Digital health technologies present a promising approach to give new insights into fatigue.

The aim of this study is to use digital health technologies, real-time self-reports, and qualitative interview data to investigate how fatigue is experienced over time in participants with myeloma, long COVID, heart failure, and in controls without problematic fatigue. Objectives are to understand which sensed parameters add value to the characterisation of fatigue and to determine whether study processes are feasible, acceptable, and scalable.

Methods and Analysis

An ecological momentary assessment study will be carried out over two or four weeks (participant defined). Individuals with fatigue relating to myeloma (n=10), heart failure (n=10), long COVID (n=10) and controls without problematic fatigue or a study condition (n=10) will be recruited. ECG patches will measure heart rate variability, respiratory rate, body temperature, activity, and posture. A wearable bracelet accompanied by environment beacons will measure physical activity, sleep, and room location within the home. Self-reports of mental and physical fatigue will be collected via smartphone app four times daily and on-demand. Validated fatigue and affect questionnaires will be completed at baseline and two weeks. End of study interviews will investigate experiences of fatigue and study participation. A feedback session will be offered to participants to discuss their data.

Data will be analysed using multilevel modelling and Machine Learning. Interviews and feedback sessions will be analysed using content or thematic analyses.

For peer review only - http://bmjopen.bmj.com/site/about/guidelines.xhtml

Ethics and dissemination: Approved by East of England – Cambridge East Research Ethics Committee (22/EE/0261). Results will be disseminated in peer reviewed journals and at international conferences.

Registration: Clinicaltrials.gov NCT05622669

Strengths and Limitations of this Study

- Integrates granular data from digital sensing technologies with participant self-reports and in-depth qualitative data
- Will use artificial intelligence techniques alongside multilevel modelling to detect patterns within temporal data
- Allows participants to reflect on their sensed data during bespoke feedback sessions, using qualitative data from these sessions to inform data interpretation
- Is not powered to quantify significant differences in fatigue ratings between groups of participants with different conditions

For peer review only

For peer review only - http://bmjopen.bmj.com/site/about/guidelines.xhtml

Introduction

Fatigue is a major clinical problem and is prevalent in most chronic medical conditions. Problematic fatigue is experienced by up to 98% of individuals with myeloma (1), around 50% of those with heart failure (2) and over 70% of those with long COVID (3). Fatigue is a multifaceted, fluctuating symptom which embodies biological, biochemical, physiological, psychological, emotional, and behavioural processes (4). Fatigue is distressing and causes reduced quality of life (5), and diminished economic productivity (6).

Fatigue is considered as a single, poorly defined symptom, but fatigue as a symptom is likely to encapsulate multidimensional experiences and fatigue is unlikely to be a single entity. There is no one widely accepted definition of fatigue, but fatigue is often described as “extreme and persistent mental and/or physical tiredness, weakness or exhaustion”(7) Other definitions encompass the negative impact that fatigue can have on the individual, for example, an “overwhelming feeling of sustained exhaustion that is debilitating and interferes with an individual’s ability to function and perform activities”(8).

There are likely to be specific patterns or characteristics of fatigue that vary according to the underlying mechanisms. For example, Powell et al. showed that individuals with multiple sclerosis (MS) were more likely to have fatigue that peaked in the afternoon, that came on more suddenly, and that was more likely to be present after physical activity compared to healthy controls (9).

People with long COVID commonly describe triggers that worsen fatigue, such as physical activity, stress, and sleep disturbance (10,11). In people with heart failure, tissue hypoperfusion and a mismatch between catabolic and anabolic processes can cause skeletal myopathy (12). This is thought to contribute to fatigue, particularly during physical exertion, and fatigue is often experienced alongside breathlessness (12). Fatigue is a common side effect of myeloma therapies, and during first treatment for myeloma, targeted medicines,

For peer review only - http://bmjopen.bmj.com/site/about/guidelines.xhtml

chemotherapy agents, and steroids are often given in cycles (13). Fatigue is likely to vary during treatment cycles (14), but this is under-explored. Specific patterns of fatigue in myeloma, heart failure and long COVID are under-researched.

Despite its high prevalence, fatigue is poorly understood (15). Fatigue research can be challenging because fatigue is changeable, subjective, and influenced by a wide range of individual, environmental, and external factors. Fatigue also poses clinical challenges. When individuals present to clinicians with undifferentiated "tiredness", standard diagnostic tests are usually unhelpful (16,17). There are limited personalised treatment options for fatigue and fatigue often remains untreated (18).

Ecological momentary assessment (EMA) (19) is a method that is primarily focussed on the investigation of within-person dynamic processes with high levels of ecological validity, using relatively intensive data collection techniques that monitor phenomena in real-time, or close to real-time, whilst participants undertake their usual daily activities (19). EMA methods have been used to gain insights into fatigue in several chronic medical conditions including renal failure(20) , MS (9)and cancer (21). EMA studies have the advantage of minimising participant recall bias and highlighting temporal variations within data (19). Few EMA studies have attempted to make comparisons between individuals with different clinical diagnoses (22). Furthermore, advances in digital health technologies offer new opportunities to combine participant self-reports of symptoms with real-time objective physiological and environmental data.

This paper describes the protocol for an EMA study of fatigue. Digital health technologies will be used to capture in-depth objective physiological, activity, self-report, and environmental measurements from individuals with myeloma, long COVID, heart failure, and a control group without these conditions. Cancers, infectious diseases, and cardiovascular

For peer review only - http://bmjopen.bmj.com/site/about/guidelines.xhtml

diseases account for a significant global disease burden (23). The exemplar conditions were chosen because they involve fatigue as a core or highly prevalent symptom, and because they are likely to involve different underlying mechanisms of fatigue, and different patterns of fatigue. They are also conditions in which we expect to see fluctuations in the fatigue experience over time that might be observable, predictable and explainable (for example, according to medication cycle in myeloma, relating to post-exertional malaise in long COVID or activity levels in heart failure).

The aim of the study is to measure fatigue within individuals over time, to quantify variations in fatigue levels within individuals, and to describe relationships between patient-reported fatigue, patient-reported triggers for fatigue, and sensed parameters including activity levels, sleep, heart rate variability, and other physiological parameters. Physiological parameters such as heart rate variability could give important insights into the fatigue experience. For example, Heart Rate Variability (HRV) is the fluctuation in time intervals between each heartbeat (24) and is linked to autonomic nervous system activation (25). Heart rate variability can give insights into emotions and stress (25). It has been suggested that HRV could be linked to fatigue severity in chronic fatigue syndrome (26). HRV has also been studied as a marker of driver fatigue (27). In this study-qualitative and quantitative data will be combined to investigate differences in the fatigue experience within and between individuals over time.

This study is exploratory and will test the feasibility, acceptability and practicalities of deploying the digital technologies and of the study procedures with a view to conducting a large-scale investigation into replicable, measurable, quantitative differences in fatigue patterns between groups of individuals with different medical conditions. The study will also develop artificial intelligence (AI) algorithms and explore the value of AI techniques in analysing and interpreting EMA study data.

For peer review only - http://bmjopen.bmj.com/site/about/guidelines.xhtml

Methods

Study design and setting

This EMA study will be conducted remotely, enrolling participants from anywhere within the United Kingdom (UK). Participants will self-rate their physical and cognitive fatigue four times daily and on demand over two or four weeks (according to participant preference). The study is an exploratory feasibility study, and the primary objective is to use both quantitative and qualitative methods to characterise lived experiences of fatigue and to explore temporal patterns in fatigue within individuals with different medical conditions.

Outcomes

The primary outcome is descriptive and concerns how patient reports of fatigue change over time, and the temporal relationships between fatigue, self-reported triggers for fatigue, and sensed parameters (see below).

Secondary outcomes relate to the feasibility of the study procedures. Secondary outcomes include ability to recruit eligible participants at the required rate; attrition rate; suitability of the study questionnaires and patient reported outcome measures (PROMS); acceptability and desirability of the study procedures; usability of the technologies; logistics of remote study assessments and data collection; and potential predictors of missing self-report data during the EMA study.

Sample Size

The study will aim to enrol 40 participants, including 10 participants with myeloma, 10 with heart failure, 10 with long COVID, and 10 control participants. It may be the case that the sample sizes in the groups are not evenly matched. This will be determined by participant interest and response. It is also possible that some participants provide limited data or have high levels of missing data (for example, due to sensor failure or poor adherence to EMA ratings). The research team may decide to recruit additional participants into a group in which

For peer review only - http://bmjopen.bmj.com/site/about/guidelines.xhtml

participants have high levels of missing data, where missing data precludes meaningful insights into the participant's fatigue experience, and this will be judged during the study by the lead researcher.

This is a feasibility study without a quantitative primary outcome measure. As such, a formal sample size calculation is not required, however the sampling design has been chosen to sufficiently capture the expected within-day variability in fatigue following discussions with patient partners. In parallel group pilot trials, a sample size of 12 participants per group is suggested as a "rule of thumb" that will give adequate data to inform future definitive studies (28). The EMA study design employed here differs from traditional parallel group studies in that multiple observations are taken in the same individual over time, allowing for detailed within person descriptions of the phenomenon and temporal relationships/associations. In this study, granular patient reported outcome data will be combined with rich qualitative data. The estimate of ten patients per group is a pragmatic one that considers the primary study objective, researcher capacity, study duration, number of daily EMA ratings, and availability of sensors and other technical equipment.

Eligibility Criteria

Inclusion and exclusion criteria are listed in Table 1. Eligible participants are adults with fatigue (considered by them to be worse than "normal tiredness") related to myeloma, long COVID, or heart failure, and control group participants without a study condition who do not experience problematic fatigue (worse than "normal tiredness"). Participants will not be given a specific definition of fatigue at the start of the study and no cut-off points will be used for the severity of fatigue or its impact on function. Instead, the experience of fatigue will be documented in detail for every participant using the combination of validated, sensed and self-report measures described below.

Table 1: Inclusion and Exclusion Criteria

For peer review only - http://bmjopen.bmj.com/site/about/guidelines.xhtml

For peer review only

Group allocation	Inclusion criteria	Exclusion criteria
All participants	Willing to participate in intermittent ecological momentary assessments of fatigue, to wear an electrocardiogram (ECG) patch, to provide questionnaire responses and to participate in an end of study interview	Difficulty communicating in English<br>Adults lacking capacity to consent<br>Under 18 years of age<br>Declines to participate<br>Under investigation for or starting treatment for an endocrine, metabolic, or thyroid condition where the participant has not been established on a stable therapeutic dose of a licensed therapy for that condition<br>A confirmed diagnosis of sleep apnoea or narcolepsy<br>HADs depression and anxiety score at baseline greater than 8 on the depression questions (29), which might indicate untreated or undertreated depression<br>Shift work that involves overnight working between the hours of 9pm and 9am
Group A, individuals with myeloma	A confirmed diagnosis of myeloma<br>Has experienced fatigue that is perceived by the participant to be worse than "normal tiredness" and that they associate with myeloma or treatment for myeloma	Uncontrolled hypercalcaemia<br>Current or previous diagnosis of heart failure or long COVID<br>An active primary cancer diagnosis other than myeloma
Group B, Individuals with heart failure	A formal diagnosis of heart failure<br>All stages of heart failure and all aetiologies and with no specific ejection fraction cut-off<br>Has experienced fatigue that is perceived by the participant to be worse than "normal tiredness" and that they associate with their cardiac disease or its treatment	A current or previous diagnosis of myeloma or long COVID<br>Active cancer
Group C, Individuals with long COVID	Experiencing fatigue that is perceived by the participant to be worse than "normal tiredness", with or without other physical or psychological symptoms that developed during or after an infection consistent with COVID-19<br>The fatigue (plus or minus any other symptoms) has continued for greater or equal to 12 weeks, and is not explained by an alternative diagnosis	A current or previous diagnosis of myeloma or heart failure<br>Active cancer
Group D, control group	Individuals aged 18 years or over without the disease conditions specified in Groups A to C	Presence of myeloma or another active cancer, heart failure, or long COVID<br>One or more chronic medical conditions which are unstable, poorly controlled, AND perceived by the individual to be causing fatigue<br>Persistent or severe fatigue symptoms that are perceived by the individual to be worse than "normal tiredness"<br>Taking sedating medications to manage anxiety or insomnia including but not limited to benzodiazepines or "Z" drugs, zopiclone, zolpidem, and others in this British National Formulary Class

For peer review only

For peer review only - http://bmjopen.bmj.com/site/about/guidelines.xhtml

Recruitment and consent

Eligible patients with myeloma will be invited to participate by clinicians from the haematology clinic at University College London Hospital. Eligible patients with heart failure will be invited to participate by clinicians working in NHS Grampian's heart failure nursing service. Participants with heart failure will also be recruited from General Practices in NHS Grampian, facilitated by the NHS Research Scotland Primary Care (NRS Primary Care) network.

Participants with myeloma, heart failure, or long COVID who took part in previous focus groups as part of this project and who have consented to being contacted about future related research will be invited to participate by email. Relevant charity and patient advocacy organisations will also be invited to share study information leaflets with their members.

Control group participants will be recruited via staff mailing lists at collaborating academic institutions, patient involvement groups, and from community walking organisations.

The study may be advertised on X (formerly known as Twitter) if recruitment targets are not achieved via other routes.

Individuals identified by NHS clinicians will be handed an invitation letter, information sheet, reply slip and reply-paid envelope by their clinicians and asked to contact the study team if they are interested in participating. Those recruited by General Practices will be identified by searches of electronic medical records and sent invitation packs by post. Those invited by email will receive the participant information sheet and invitation letter as an attachment and will be asked to reply by email to the study team to indicate their interest.

Interested individuals will have an initial telephone or Teams video call, during which a researcher will explain the study and answer any questions. Eligibility screening will be conducted, and for eligible participants who wish to participate, audio-recorded verbal

For peer review only - http://bmjopen.bmj.com/site/about/guidelines.xhtml

consent will be taken. A paper consent form will be initialled and signed by the researcher and a copy will be sent to the participant. A copy of the participant consent form is provided in Supplementary file 1.

Participants will be offered a £50 voucher as a token of thanks.

Participant timeline and Data Collection Methods

The participant timeline is summarised in Figure 1.

Baseline Assessments

At study baseline, participants will complete the Mental and Physical State and Trait Energy and Fatigue Scales (STEF) parts one and three (30–32); Positive and Negative Affect Scales (PANAS) for "past few weeks" time frame (33) and the Modified Fatigue Impact Scale (MFIS)(34). These questionnaires were chosen as they have been validated and administered together, they will give insights into both physical and mental fatigue, "usual" fatigue (as a complement to the EMA measures), emotions, and the impact of fatigue on the individual.

Sensors

Participants will be posted a study kit containing an android smartphone with data SIM, an ECG patch (Vital Patch, Vital Connect Inc, California, USA), a wearable bracelet containing accelerometer accompanied by four environment beacons (Panoramic Digital Health SAS, Grenoble France).

A wide range of technical options were considered to capture the data required in this study. A selection of these and the factors that were considered when choosing the technologies are presented in Supplementary file 2. The technologies were discussed within the whole study team and consensus was reached about the technologies to deploy.

The VitalPatch ECG is an approved medical device and will be worn for the first seven days of the study. The patch measures respiratory rate, a single lead ECG with RR interval, heart rate parameters (including heart rate variability), body temperature, activity levels

For peer review only - http://bmjopen.bmj.com/site/about/guidelines.xhtml

(actimetry), and body position. The ECG patch communicates by Bluetooth with a dedicated app (Medibiosense Ltd, UK) pre-installed on the study phone.

The Panoramic digital health bracelet (Panoramic Digital Health SAS, Grenoble, France) is a wrist worn device containing a three-axis accelerometer, three axis magnetometer, three gyroscope, temperature sensor and atmospheric pressure sensor to measure physical activity and sleep. The bracelet is used alongside four Bluetooth beacons, which are placed within labelled positions within the participants' homes (e.g., kitchen, living room, bedroom, top of stairs). The beacons communicate with the bracelet and generate data about the wearer's proximity to each beacon, and also record temperature, sound level and light level in their location, providing environmental context to the bracelet sensor data.

EMA Assessments

Participants will use the m-path app (https://m-path.io), which is a GDPR compliant research tool developed by the Katholieke Universiteit Leuven. The app will be pre-loaded onto the study smartphone, or participants can opt to download this on their own device if they prefer. The app will be used to send participants short questionnaires four times daily to rate their cognitive fatigue and physical fatigue from zero to ten, and to note any associated triggers for improvements in or deteriorations in fatigue. The anchoring and wording of the self-rating questions is based on "state" fatigue items from the Mental and Physical State and Trait Energy and Fatigue Scale (30). A full summary of the self-rating questions is provided in text box 1.

M-path will send audible alerts to the smartphone at pre-determined intervals, spaced throughout the day and pre-agreed with the participant to fit with work or personal commitments. Participants will be able to miss self-reports and will be asked to add extra reports on-demand if they notice that their fatigue levels are better or worse than usual.

For peer review only - http://bmjopen.bmj.com/site/about/guidelines.xhtml

Text Box 1: Self-rating prompts within the m-Path app

1. Overall, how fatigued do you feel at the moment?
• I feel no fatigue, strongest feeling of fatigue ever (0-10 slider)
2. How do you feel right now with regard to your capacity to perform your typical PHYSICAL ACTIVITIES....
• I feel no fatigue – 0-10 slider – Strongest feelings of fatigue ever felt
3. How do you feel right now with regard to your capacity to perform your typical MENTAL ACTIVITIES....
• I feel no fatigue – 0-10 slider – Strongest feelings of fatigue ever felt
4. Is your fatigue better, the same, or worse than usual?
CONDITIONAL
• IF the same – no further questions
• IF better – question about factors making better
• IF worse – question about factors making worse
Factors making better:
Do you think that any of the following might have affected your current levels of fatigue (select all that apply)?
• Exercise or physical activity within the last day
• Exercise or physical activity more than a day ago
• Taking a medicine (please specify)
• Taking rest
• Taking caffeine
• Less emotions or stress
• My mood
• Spending time with other people
• The weather
• Quiet time
• Something else (please specify) – free text option
Factors making worse:
Do you think that any of the following might have affected your current levels of fatigue (select all that apply)?
• Exercise or physical activity within the last day
• Exercise or physical activity more than a day ago
• Taking a medicine (please specify)
• Emotions or stress
• My mood
• Tasks or work relating to my employment
• Tasks or work relating to my home life
• Spending time with other people
• The weather
• Noise
• Other (please specify)

End of study assessments

Participants complete all baseline questionnaires again after two weeks and participate in an end of study qualitative interview, conducted according to a schedule (Supplementary file 3).

For peer review only - http://bmjopen.bmj.com/site/about/guidelines.xhtml

They are invited to participate in an optional feedback and “sense-making” session, during which a researcher provides them with feedback on the data provided and any interesting features of the data are discussed. Feedback sessions are audio-recorded and used in analyses.

Any adverse events relating to the use of technology or participating in the study will be recorded. Any serious adverse events will be reported to the sponsor.

Data Management and analysis

ECG patch data will be downloaded manually from the phone by the research team when the phone is returned. Raw encrypted data from the Panoramic bracelets are also downloaded directly from the bracelets by the research team, where data are decrypted, quality checked and analysed. Periods of non-wear are detected using the temperature sensor and accelerometer. The raw accelerometer data is processed using the open source OxWearables algorithms to measure step count per minute (35), sleep and level of activity (36). The beacon data is processed to label the processed bracelet sensor data with location in the home, by identifying the beacon with the highest Bluetooth received signal strength indicator (RSSI). Visual inspection of the output of these algorithms alongside the VitalPatch output can be viewed in a data annotator to identify features of interest in the data including timing of getting out of bed, timing of any stair climb, and timing of any period of immobility following movement in the home. m-Path app data are downloaded from an on-line study dashboard

Summary data can be generated from the Panoramic digital health platform, including: total sleep time, step count per hour over the day, time spent and activity in each room per day, time spent and activity when away from home. More granular data can also be determined including time taken to get out of bed, number of room transitions per day, average daily time climbing stairs (where applicable), cadence for walking, and total period of immobility following movements within the home, and whether the participant gets out of bed when they

For peer review only - http://bmjopen.bmj.com/site/about/guidelines.xhtml

wake up at night (eg: to go to toilet or kitchen) or stay in bed. Data on directional change (e.g. turning/changing walking direction), change in height (e.g.: going up stairs, walking up slopes) can also be generated

Patient feedback reports will be created manually, identifying potentially interesting or unexplained features from self-report and sensed data, for example particularly high or low self-reported fatigue scores, or rapid changes in fatigue scores. Visualisations will be produced (format and content will be iterative and refined during the study based on participant feedback). Visualisations will show fatigue self-rating scores superimposed on charts showing date, time, activity levels, sleep patterns, and location within the home or being out of the home. The reports will also provide short narrative summaries about the participants' fatigue self-ratings and patterns that are being seen within the data. Feedback sessions with DH (Professor of Digital Health), CS (Professor of Human-Computing Interaction), or RA (academic General Practitioner) will be audio-recorded and used to give insights into the face validity of the sensed data and the fatigue experience more broadly. Participants will not be given medical or self-management advice.

Artificial intelligence (AI) algorithms will be created by VM and SG (University of Cambridge) to automatically handle multi-variety/multidimensional data, detect symptom fluctuation over time, and identify patterns, trends, and groups. New algorithms using machine learning (ML) will be developed and tested to understand patterns of fatigue and relationships between sensed parameters. Within and between person and within and between group differences will be analysed using multi-level modelling and machine learning. Despite the limited number of participants to be enrolled in this study, the high frequency of both the sensor data and EMA responses makes it possible to build exploratory AI models that link features derived from the sensor data and the EMA responses. Models will be exploratory but

For peer review only

For peer review only - http://bmjopen.bmj.com/site/about/guidelines.xhtml

could identify potential relationships between the sensor data and EMA responses that could inform the design of future studies and larger scale AI models

Qualitative data from interview transcripts will be analysed using Framework and thematic analysis.

Multiple investigators will be involved in the analyses. Pseudonymised data will be shared using a data sharing platform approved by the University of Aberdeen. The final dataset will be available to investigators whose proposed use of the data has been approved by the Research Ethics Committee and who are parties to the project data sharing agreement.

Patient and Public Involvement

Six on-line focus groups were conducted with individuals with fatigue (qualitative results will be reported separately). Participants with experience of a study condition gave insights into their lived experience of fatigue and their thoughts about using digital technologies to measure aspects of the fatigue experience. The results from the focus groups directly informed study design, including study duration. Some participants would find four weeks of data collection onerous, whereas for others, two weeks of data collection would be insufficient to capture perceived patterns in their fatigue (e.g. during myeloma treatment cycles).

The focus groups also led to the inclusion of the participant feedback session. It was important to participants that their sensed and self-reported data were explained to them and that they were given the opportunity to reflect on this data with an experienced researcher.

A patient partner with myeloma liaised with the study team during the preparation of the study documents and gave feedback on the protocol and patient information sheet.

Potential Implications

For peer review only - http://bmjopen.bmj.com/site/about/guidelines.xhtml

This is an exploratory feasibility study but an ambition for the work is that we start to identify discrete patterns of fatigue in different individuals. Identifying different fatigue signatures could lead to more effective classification of fatigue. Ultimately, a fatigue classification system could help with diagnosis of unexplained or undifferentiated fatigue and to tailor different management approaches.

The feasibility study will also provide practical details about the usability, reliability, acceptability and utility of the sensors. Feedback sessions with the participants will help to gauge face validity of the measurements, and which, if any, sensed parameters are meaningful to people with fatigue. Objective digital measurements that correlate well with subjective experiences in people with fatigue could enhance clinical trials of drug and non-drug treatments for fatigue both by providing objective inclusion criteria, and endpoints for efficacy. The measurements might also have implications for fatigue self-management. Digital measurements could be used to objectively identify factors (e.g. activity patterns, sleep patterns, diurnal variations) that improve or worsen fatigue levels, and that could potentially be modified. The measurements may be used in the future to help to inform pacing, goal setting, and other self-management approaches to fatigue management."

Ethics and dissemination

The study is sponsored by the University of Aberdeen. It was approved by the East of England – Cambridge East Research Ethics Committee (22/EE/0261). The study team are interdisciplinary researchers spanning primary care (clinical), health psychology, engineering, medical physics, human computing interaction, and artificial intelligence. Results will be disseminated in a range of peer reviewed journals spanning these disciplines, and at international conferences.

For peer review only - http://bmjopen.bmj.com/site/about/guidelines.xhtml

Study Status

Participant recruitment commenced on 7th December 2022 and was completed in December 2023. Forty participants were consented. Feedback sessions with the researchers are ongoing and new data are still being generated from these sessions. Data analysis is in the early stages. Data collection is expected to be complete by September 2024. The approved protocol is version 2.0, 8th December 2022.

Funding Statement

This work is being funded by Engineering and Physical Sciences Research Council (EPSRC). Grant reference EP/W003228/1. Dr Rosalind Adam is funded by a Chief Scientist Office (Scotland) Senior Clinical Academic Fellowship (reference SCAF/18/02). Neither the funders nor the sponsor had any input into the design of this study and will have no role in data analysis.

Acknowledgements

The research team would like to thank all patient participants in our on-line focus groups for their input into the design of this study, and to the National Health Service clinicians who are assisting with participant identification and study invitations.

Competing Interests

Derek Hill, Professor of Digital Health at UCL, is also founder and CEO of Panoramic Digital Health, a company that is providing technology for this study. This potential conflict of interest is documented in accordance with UCL Disclosure of Conflict and Declaration of Interest Policy and has been reviewed by Sponsor.

None of the other collaborators have any conflicts to declare.

For peer review only

Author Contributions

Rosalind Adam contributed to: conceptualisation; funding acquisition, methodology, writing – original draft preparation, and writing – review and editing.

Yojana Lotankar contributed to project administration and writing – review and editing.

Corina Sas contributed to conceptualisation, funding acquisition, and writing – review and editing.

Daniel Powell contributed to methodology and writing – review and editing.

Veronica Martinez contributed to conceptualisation, funding acquisition, and writing – review and editing.

Stephen Green contributed to writing - review and editing.

Jonathan Cooper contributed to conceptualisation, funding acquisition, and writing – review and editing.

Katherine Bradbury contributed to conceptualisation, funding acquisition, and writing – review and editing.

Jonathan Sive contributed to writing – review and editing.

Derek Hill contributed to conceptualisation, funding acquisition, and writing – review and editing.

Figure 1: Participant timeline and flow chart

References

1. Ramsenthaler C, Kane P, Gao W, Siegert RJ, Edmonds PM, Schey SA, et al. Prevalence of symptoms in patients with multiple myeloma: a systematic review and meta-analysis. Eur J Haematol [Internet]. 2016 Nov 1 [cited 2022 Jul 15];97(5):416–29. Available from: https://pubmed.ncbi.nlm.nih.gov/27528496/

For peer review only - http://bmjopen.bmj.com/site/about/guidelines.xhtml

2. Barnes S, Gott M, Payne S, Parker C, Seamark D, Gariballa S, et al. Prevalence of symptoms in a community-based sample of heart failure patients. J Pain Symptom Manage [Internet]. 2006 Sep;32(3):208–16. Available from: http://www.ncbi.nlm.nih.gov/pubmed/16939845

3. Prevalence of ongoing symptoms following coronavirus (COVID-19) infection in the UK - Office for National Statistics [Internet]. [cited 2023 Feb 2]. Available from: https://www.ons.gov.uk/peoplepopulationandcommunity/healthandsocialcare/conditionsanddiseases/bulletins/prevalenceofongoingsymptomsfollowingcoronaviruscovid19infectionintheuk/2february2023

4. Swain MG. Fatigue in chronic disease. Clin Sci (Lond) [Internet]. 2000 Jul;99(1):1–8. Available from: http://www.ncbi.nlm.nih.gov/pubmed/10887052

5. Kluger BM, Krupp LB, Enoka RM. Fatigue and fatigability in neurologic illnesses: proposal for a unified taxonomy. Neurology [Internet]. 2013 Jan 22;80(4):409–16. Available from: http://www.ncbi.nlm.nih.gov/pubmed/23339207

6. Finsterer J, Mahjoub SZ. Fatigue in healthy and diseased individuals. Am J Hosp Palliat Care [Internet]. 2014 Aug;31(5):562–75. Available from: http://www.ncbi.nlm.nih.gov/pubmed/23892338

7. Dittner AJ, Wessely SC, Brown RG. The assessment of fatigue. J Psychosom Res. 2004 Feb;56(2):157–70.

8. Jaime-Lara RB, Koons BC, Matura LA, Hodgson NA, Riegel B. A Qualitative Metasynthesis of the Experience of Fatigue Across Five Chronic Conditions. J Pain Symptom Manage. 2020 Jun;59(6):1320–43.

9. Powell DJH, Liossi C, Schlotz W, Moss-Morris R. Tracking daily fatigue fluctuations in multiple sclerosis: ecological momentary assessment provides unique insights. J Behav Med [Internet]. 2017 Oct;40(5):772–83. Available from: http://www.ncbi.nlm.nih.gov/pubmed/28281106

10. Ziauddeen N, Gurdasani D, O'Hara ME, Hastie C, Roderick P, Yao G, et al. Characteristics and impact of Long Covid: Findings from an online survey. PLoS One [Internet]. 2022 Mar 1 [cited 2023 Feb 2];17(3). Available from: https://pubmed.ncbi.nlm.nih.gov/35259179/

11. Sas C, Lotankar Y, Adam R, Bradbury KJ, Cooper J, Hill DL, et al. "A Switch Went off in my Whole Body": Lived Experiences of Fatigue and Post-Exertional Malaise in Long Covid. Extended Abstracts of the 2023 CHI Conference on Human Factors in Computing Systems [Internet]. 2023 Apr 19 [cited 2023 Apr 28];1–7. Available from: https://dl.acm.org/doi/10.1145/3544549.3585846

12. Clark AL. Origin of symptoms in chronic heart failure. Heart. 2006 Jan;92(1):12–6.

13. Goldman-Mazur S, Kumar SK. Current approaches to management of high-risk multiple myeloma. Am J Hematol [Internet]. 2021 Jul 1 [cited 2023 Feb 2];96(7):854–71. Available from: https://pubmed.ncbi.nlm.nih.gov/33725367/

For peer review only

For peer review only - http://bmjopen.bmj.com/site/about/guidelines.xhtml

14. Dombeck C, Swezey T, Gonzalez Sepulveda JM, Reeve BB, LeBlanc TW, Chandler D, et al. Patient perspectives on considerations, tradeoffs, and experiences with multiple myeloma treatment selection: a qualitative descriptive study. BMC Cancer [Internet]. 2023 Dec 1 [cited 2023 Feb 9];23(1). Available from: /pmc/articles/PMC9850680/

15. Matura LA, Malone S, Jaime-Lara R, Riegel B. A Systematic Review of Biological Mechanisms of Fatigue in Chronic Illness. Biol Res Nurs. 2018 Jul;20(4):410-421.

16. Kitai E, Blumberg G, Levy D, Golan-Cohen A, Vinker S. Fatigue as a first-time presenting symptom: management by family doctors and one year follow-up. Isr Med Assoc J [Internet]. 2012 Sep;14(9):555–9. Available from: http://www.ncbi.nlm.nih.gov/pubmed/23101419

17. Gialamas A, Beilby JJ, Pratt NL, Henning R, Marley JE, Roddick JF. Investigating tiredness in Australian general practice. Do pathology tests help in diagnosis? Aust Fam Physician [Internet]. 2003 Aug;32(8):663–6. Available from: http://www.ncbi.nlm.nih.gov/pubmed/12973880

18. Vaes AW, Goërtz YMJ, van Herck M, Beijers RJHCG, van Beers M, Burtin C, et al. Physical and mental fatigue in people with non-communicable chronic diseases. Ann Med [Internet]. 2022 [cited 2023 Apr 5];54(1):2522–34. Available from: https://pubmed.ncbi.nlm.nih.gov/36111684/

19. Shiffman S, Stone AA, Hufford MR. Ecological Momentary Assessment. Annu Rev Clin Psychol. 2008 Apr 1;4(1):1–32.

20. Abdel-Kader K, Jhamb M, Mandich LA, Yabes J, Keene RM, Beach S, et al. Ecological momentary assessment of fatigue, sleepiness, and exhaustion in ESKD. BMC Nephrol [Internet]. 2014 Feb 6 [cited 2023 Feb 3];15(1). Available from: https://pubmed.ncbi.nlm.nih.gov/24502751/

21. Kampshoff CS, Verdonck-de Leeuw IM, van Oijen MG, Sprangers MA, Buffart LM. Ecological momentary assessments among patients with cancer: A scoping review. Eur J Cancer Care (Engl). 2019 May;28(3):e13095.

22. Perski O, Keller J, Kale D, Asare BYA, Schneider V, Powell D, et al. Understanding health behaviours in context: A systematic review and meta-analysis of ecological momentary assessment studies of five key health behaviours. Health Psychol Rev. 2022 Oct 2;16(4):576–601.

23. Naghavi M, Ong KL, Aali A, Ababneh HS, Abate YH, Abbafati C, et al. Global burden of 288 causes of death and life expectancy decomposition in 204 countries and territories and 811 subnational locations, 1990–2021: a systematic analysis for the Global Burden of Disease Study 2021. The Lancet. 2024 Apr;

24. Ni Z, Sun F, Li Y. Heart Rate Variability-Based Subjective Physical Fatigue Assessment. Sensors. 2022 Apr 21;22(9):3199.

25. Csathó Á, Van der Linden D, Matuz A. Change in heart rate variability with increasing time-on-task as a marker for mental fatigue: A systematic review. Biol Psychol. 2024 Jan;185:108727.

For peer review only - http://bmjopen.bmj.com/site/about/guidelines.xhtml

26. Escorihuela RM, Capdevila L, Castro JR, Zaragozà MC, Maurel S, Alegre J, et al. Reduced heart rate variability predicts fatigue severity in individuals with chronic fatigue syndrome/myalgic encephalomyelitis. J Transl Med. 2020 Dec 6;18(1):4.

27. Lu K, Sjörs Dahlman A, Karlsson J, Candefjord S. Detecting driver fatigue using heart rate variability: A systematic review. Accid Anal Prev. 2022 Dec;178:106830.

28. Julious SA. Sample size of 12 per group rule of thumb for a pilot study. Pharm Stat [Internet]. 2005 Oct 1 [cited 2022 Jul 15];4(4):287–91. Available from: https://onlinelibrary.wiley.com/doi/full/10.1002/pst.185

29. Zigmond AS, Snaith RP. The Hospital Anxiety and Depression Scale. Acta Psychiatr Scand. 1983 Jun;67(6):361–70.

30. O'Connor P.J. Mental and physical state and trait energy and fatigue scales: [unpublished manual]. University of Georgia., 2006.

31. Boolani A, Manierre M. An exploratory multivariate study examining correlates of trait mental and physical fatigue and energy. Fatigue. 2019 Jan 2;7(1):29–40.

32. Cook DB, O'Connor PJ, Lange G, Steffener J. Functional neuroimaging correlates of mental fatigue induced by cognition among chronic fatigue syndrome patients and controls. Neuroimage. 2007 May;36(1):108–22.

33. Watson D, Clark LA, Tellegen A. Development and validation of brief measures of positive and negative affect: The PANAS scales. J Pers Soc Psychol. 1988;54(6):1063–70.

34. Fisk JD, Ritvo PG, Ross L, Haase DA, Marrie TJ, Schlech WF. Measuring the Functional Impact of Fatigue: Initial Validation of the Fatigue Impact Scale. Clinical Infectious Diseases. 1994 Jan 1;18(Supplement_1):S79–83.

35. Small SR, Chan S, Walmsley R, Fritsch L von, Acquah A, Mertes G, et al. Development and Validation of a Machine Learning Wrist-worn Step Detection Algorithm with Deployment in the UK Biobank. medRxiv [Internet]. 2023 Jan 1;2023.02.20.23285750. Available from: http://medrxiv.org/content/early/2023/02/22/2023.02.20.23285750.abstract

36. Doherty A, Smith-Byrne K, Ferreira T, Holmes M V, Holmes C, Pulit SL, et al. GWAS identifies 14 loci for device-measured physical activity and sleep duration. Nat Commun. 2018 Dec 10;9(1):5257.

For peer review only

For peer review only - http://bmjopen.bmj.com/site/about/guidelines.xhtml

Figure 1: Participant timeline and flow chart. *Key data collection time points are highlighted in bold*

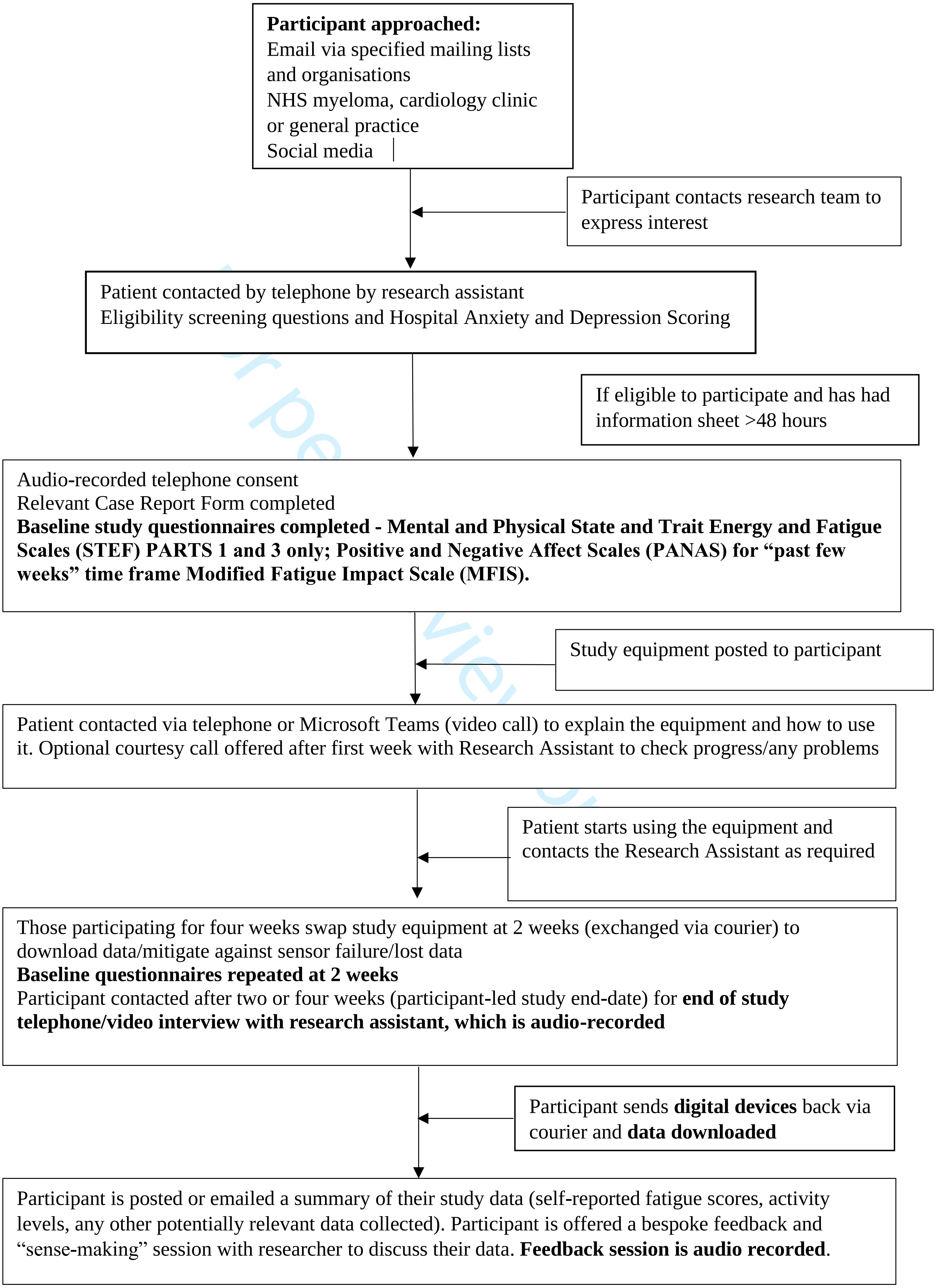

For peer review only - http://bmjopen.bmj.com/site/about/guidelines.xhtml

UNIVERSITY OF ABERDEEN

Participant identification number

RECORD OF PARTICIPANT'S VERBAL CONSENT

Understanding Patterns of Fatigue in Health and Disease

The following is a telephone script and record of verbal consent for participants who indicate to the researchers that they would like to participate in the study. A copy of this consent form will be scanned or photocopied and sent to the participant via email or by post (according to their preference). Researcher introduces themself and the study and checks whether it is an appropriate time to conduct the consent process. Researcher checks participant's name and asks permission to audio-record the consent process. Audio-recorder is then turned on.

Chief Investigator: Dr Rosalind Adam

Researcher initials

1. I confirm that I have read and understand the information sheet (version .... date ....) for the above study, have had the opportunity to ask questions, and have had these answered satisfactorily. ☐
2. I understand that my participation is voluntary and that I am free to withdraw at any time, without giving any reason, without my medical care or legal rights being affected. Only anonymised data collected up until the point of withdrawal may still be used in analysis. ☐
3. I agree to provide data collected via a wearable patch and a wrist worn device ☐
4. I agree to provide ratings of my fatigue via a smartphone app ☐
5. I agree to taking part in an interview with a researcher at the end of the study ☐
6. I agree to my interview being audio-recorded. I understand that my recording will be kept confidential and that anonymous quotations from recordings may be used in presentations and publications. ☐
7. I agree to take part in a "feedback session" after my data have been analysed. I agree to this session being audio recorded. I understand that my recording will be kept confidential and that anonymous quotations from this session may be used in publications and presentations Yes ☐ No ☐

For peer review only - http://bmjopen.bmj.com/site/about/guidelines.xhtml

8. I agree that Information about me can be stored on University of Aberdeen computer servers. ☐

9. I agree that my data can be shared with the collaborating investigators from University College London, University of Cambridge, University of Lancaster, Southampton University, University of Glasgow and Panoramic Digital Health. I understand that identifiable information such as contact details will only be shared when it is necessary for a research team member from one of these organisations to contact me about the study (for example, in the feedback session). ☐

10. I agree that researchers can contact my general practitioner in the case that any incidental findings are noted during the study (for example, ECG changes that the researchers were not seeking to find or investigating, but that have been noted by researchers and which might be medically relevant) ☐

11. I understand that data collected during the study may be looked at by individuals from the University of Aberdeen, the regulatory authorities or from NHS, where it is relevant to my taking part in this research. I give permission for these individuals to have access to this data. ☐

11. I would like to be invited to take part in future ethically reviewed and approved research related to this study. I understand identifiable contact information will be kept after the end of this study and this information will be held in accordance with data protection regulation. Yes ☐ No ☐

12. I agree to take part in the above study ☐

(If Participant agrees to participate, researcher asks the participant to confirm their full name and signs and dates below)

Name of Participant ____

______________________	______________________	______________________
Name of Researcher	Signature of Researcher	Date and Time

For peer review only - http://bmjopen.bmj.com/site/about/guidelines.xhtml

For peer review only - http://bmjopen.bmj.com/site/about/guidelines.xhtml

Supplementary data file 2: Selecting technologies for the study

Selection of the technologies and platforms considered as part of the procurement process

- Philips health band https://www.usa.philips.com/healthcare/product/HC422210064081/philips-health-band#specifications
- Philips Actiwatch 2
- M-Path https://m-path.io/landing/
- PRO Diary CamNtech PRO-Diary - CamNtech
- LifeData experience platform LifeData Experience Sampling App - LifeData Experience Sampling App for Research (lifedatacorp.com)
- MediBiosense ECG patch technology:
- Movisens EcgMove4, Move 4, SensorTrigger, EdaMove 4, LKightMove4 Products - movisens GmbH

Key factors that were considered when choosing technologies to capture project data

- Range of data parameters that can be captured or measured
- Study team experience and knowledge of the technology and successful deployment in other projects
- Cost
- Support infrastructure for technical problems
- Ease of data download/supports data flow within the trial, maintaining privacy and security
- GDPR compliance
- Accuracy (e.g. consensus that wrist-worn heart rate monitoring lacked the accuracy required for our study)
- Ease of use/setting up for end user/requirement for participant internet connectivity for successful deployment
- Ability to interact with raw data from devices/apply and test different algorithms to raw data

TOPIC GUIDE FOR END OF STUDY INTERVIEWS

Understanding Patterns of Fatigue in Health and Disease

Interviews will be directed to some extent by the participants. This document sets out topics that the interviewer will aim to cover. It will be used flexibly.

For peer review only

INTRODUCTIONS

- Introductions and reminder that interview can be paused or stopped at any time. Audio-recording device activated.
- In this interview, we would like to ask you about your experiences of fatigue over the study period and your opinions about taking part in the study and using the study devices.

GENERAL CONTEXT

- How have you been in general over the past two weeks?
- Have these been typical weeks for you (prompts might include usual activity levels/work/typical levels of fatigue)

FATIGUE EXPERIENCE

- Tell me about (any) fatigue that you have experienced over the last two weeks? Prompts might include: severity, how often fatigue was experienced, whether fatigue was mainly mental/physical and how it felt in the body.

If fatigue was experienced (control group participants may not have experienced fatigue):

- Do you remember any specific day to have been particularly more difficult or particularly good (in terms of fatigue)?
- Can you think of any triggers or things that might have worsened your fatigue? (prompts might include: physical activity, sleep, medications, stress, treatment cycle, menstruation, weather)
- How did the fatigue impact your physical activity?
- How did the fatigue impact your cognitive ability?
- What did you do to cope with your fatigue or any things that have made the fatigue better/helped?

FEASIBLITY

We would like to will discuss more specifically about some of the devices and ways that we have collected your information throughout the study.

- Did you face any difficulties using any of the devices? (Prompted to describe these, prompts might include app set up, ECG patch set up, use of app, whether the devices were uncomfortable or hindered activities/movements).

1. Panoramic Digital Health Bracelet and Bluetooth Tags
   - What did you like most about using the bracelet?
   - What did you not like about the device?
   - How could it be improved?
   - How easy or difficult it was to set up and use?

2. ECG Patch
   - What did you like about using the ECG Patch?
   - What did you not like, and could be different?
   - How easy or difficult was it to use?

3. Daily fatigue scores (m-Path)

   - Tell me about your experiences of using the app to enter fatigue scores. Prompts might include:
     - How easy/difficult it was to use the app
     - How easy/difficult was it to enter the fatigue scores? Why?
     - Whether the number and timings of the ratings were convenient/inconvenient, whether the ratings were too frequent/not frequent enough/just right to capture the difference/variability in fatigue
     - Whether the questions were easy to understand
     - Can you think of any other questions that could better capture your daily fatigue? What and why?
     - Is there anything that you did you not like about scoring our fatigue daily? What could be done better?
     - How convenient was it to use the app?
     - Would you rather have preferred a different method to input your daily scores? How? Why?

4. We used three questionnaires at the start and end of the study (participant is reminded about the nature of the questionnaires). Did you find the questions in these questionnaires easy to understand? Do you think the questions were relevant to you?

SUMMING UP

- Overall, how have you found participating in this study?
  - Any positive things about taking part
  - Any negative things about taking part
- We have covered lots of things (give brief summary); is there anything else that you would like to speak about that we haven't covered?

Participant is thanked for their time and reminded that they will have the opportunity to get a feedback session with a senior researcher to go over the data they have provided.

END of INTERVIEW

For peer review only

SPIRIT — Standard Protocol Items: Recommendations for Interventional Trials

SPIRIT 2013 Checklist: Recommended items to address in a clinical trial protocol and related documents*

Section/item	Item No	Description	Addressed on page number
**Administrative information**			
Title	1	Descriptive title identifying the study design, population, interventions, and, if applicable, trial acronym	_______1________
Trial registration	2a	Trial identifier and registry name. If not yet registered, name of intended registry	_________3______
	2b	All items from the World Health Organization Trial Registration Data Set	________3 (and in online trial registration)______
Protocol version	3	Date and version identifier	_______17________
Funding	4	Sources and types of financial, material, and other support	________18_______
Roles and responsibilities	5a	Names, affiliations, and roles of protocol contributors	________1_______
	5b	Name and contact information for the trial sponsor	_______17________
	5c	Role of study sponsor and funders, if any, in study design; collection, management, analysis, and interpretation of data; writing of the report; and the decision to submit the report for publication, including whether they will have ultimate authority over any of these activities	_______18________

For peer review only

	5d	Composition, roles, and responsibilities of the coordinating centre, steering committee, endpoint adjudication committee, data management team, and other individuals or groups overseeing the trial, if applicable (see Item 21a for data monitoring committee)	_________N/A____ _
**Introduction**			
Background and rationale	6a	Description of research question and justification for undertaking the trial, including summary of relevant studies (published and unpublished) examining benefits and harms for each intervention	_____4,5,6______ __
	6b	Explanation for choice of comparators	____5,6________ _
Objectives	7	Specific objectives or hypotheses	_____6________
Trial design	8	Description of trial design including type of trial (eg, parallel group, crossover, factorial, single group), allocation ratio, and framework (eg, superiority, equivalence, noninferiority, exploratory)	_______5,7______ _
**Methods: Participants, interventions, and outcomes**			
Study setting	9	Description of study settings (eg, community clinic, academic hospital) and list of countries where data will be collected. Reference to where list of study sites can be obtained	_______7_______
Eligibility criteria	10	Inclusion and exclusion criteria for participants. If applicable, eligibility criteria for study centres and individuals who will perform the interventions (eg, surgeons, psychotherapists)	_____8,9_______ _
Interventions	11a	Interventions for each group with sufficient detail to allow replication, including how and when they will be administered	11,12,13______
	11b	Criteria for discontinuing or modifying allocated interventions for a given trial participant (eg, drug dose change in response to harms, participant request, or improving/worsening disease)	__N/A______
	11c	Strategies to improve adherence to intervention protocols, and any procedures for monitoring adherence (eg, drug tablet return, laboratory tests)	____N/A______

For peer review only - http://bmjopen.bmj.com/site/about/guidelines.xhtml

	11d	Relevant concomitant care and interventions that are permitted or prohibited during the trial	___9,10_______
Outcomes	12	Primary, secondary, and other outcomes, including the specific measurement variable (eg, systolic blood pressure), analysis metric (eg, change from baseline, final value, time to event), method of aggregation (eg, median, proportion), and time point for each outcome. Explanation of the clinical relevance of chosen efficacy and harm outcomes is strongly recommended	___7______
Participant timeline	13	Time schedule of enrolment, interventions (including any run-ins and washouts), assessments, and visits for participants. A schematic diagram is highly recommended (see Figure)	__Figure 1_
Sample size	14	Estimated number of participants needed to achieve study objectives and how it was determined, including clinical and statistical assumptions supporting any sample size calculations	______7,8_____
Recruitment	15	Strategies for achieving adequate participant enrolment to reach target sample size	______10,11____

Methods: Assignment of interventions (for controlled trials)

Allocation:

Sequence generation	16a	Method of generating the allocation sequence (eg, computer-generated random numbers), and list of any factors for stratification. To reduce predictability of a random sequence, details of any planned restriction (eg, blocking) should be provided in a separate document that is unavailable to those who enrol participants or assign interventions	____N/A______
Allocation concealment mechanism	16b	Mechanism of implementing the allocation sequence (eg, central telephone; sequentially numbered, opaque, sealed envelopes), describing any steps to conceal the sequence until interventions are assigned	_____N/A_____
Implementation	16c	Who will generate the allocation sequence, who will enrol participants, and who will assign participants to interventions	__N/A_____
Blinding (masking)	17a	Who will be blinded after assignment to interventions (eg, trial participants, care providers, outcome assessors, data analysts), and how	___N/A_____
	17b	If blinded, circumstances under which unblinding is permissible, and procedure for revealing a participant's allocated intervention during the trial	___N/A______

Methods: Data collection, management, and analysis

Data collection methods	18a	Plans for assessment and collection of outcome, baseline, and other trial data, including any related processes to promote data quality (eg, duplicate measurements, training of assessors) and a description of study instruments (eg, questionnaires, laboratory tests) along with their reliability and validity, if known. Reference to where data collection forms can be found, if not in the protocol	______14_______
	18b	Plans to promote participant retention and complete follow-up, including list of any outcome data to be collected for participants who discontinue or deviate from intervention protocols	_____N/A_____
Data management	19	Plans for data entry, coding, security, and storage, including any related processes to promote data quality (eg, double data entry; range checks for data values). Reference to where details of data management procedures can be found, if not in the protocol	______14_______
Statistical methods	20a	Statistical methods for analysing primary and secondary outcomes. Reference to where other details of the statistical analysis plan can be found, if not in the protocol	_____14,15,16___ _____
	20b	Methods for any additional analyses (eg, subgroup and adjusted analyses)	_______15____
	20c	Definition of analysis population relating to protocol non-adherence (eg, as randomised analysis), and any statistical methods to handle missing data (eg, multiple imputation)	_____N/A___

Methods: Monitoring

Data monitoring	21a	Composition of data monitoring committee (DMC); summary of its role and reporting structure; statement of whether it is independent from the sponsor and competing interests; and reference to where further details about its charter can be found, if not in the protocol. Alternatively, an explanation of why a DMC is not needed	___N/A______
	21b	Description of any interim analyses and stopping guidelines, including who will have access to these interim results and make the final decision to terminate the trial	_____N/A_______
Harms	22	Plans for collecting, assessing, reporting, and managing solicited and spontaneously reported adverse events and other unintended effects of trial interventions or trial conduct	_____14________
Auditing	23	Frequency and procedures for auditing trial conduct, if any, and whether the process will be independent from investigators and the sponsor	_____N/A_______

For peer review only - http://bmjopen.bmj.com/site/about/guidelines.xhtml

Ethics and dissemination

Research ethics approval	24	Plans for seeking research ethics committee/institutional review board (REC/IRB) approval	__17___________
Protocol amendments	25	Plans for communicating important protocol modifications (eg, changes to eligibility criteria, outcomes, analyses) to relevant parties (eg, investigators, REC/IRBs, trial participants, trial registries, journals, regulators)	____N/A_____
Consent or assent	26a	Who will obtain informed consent or assent from potential trial participants or authorised surrogates, and how (see Item 32)	___10_____
	26b	Additional consent provisions for collection and use of participant data and biological specimens in ancillary studies, if applicable	___N/A_______
Confidentiality	27	How personal information about potential and enrolled participants will be collected, shared, and maintained in order to protect confidentiality before, during, and after the trial	_____16________
Declaration of interests	28	Financial and other competing interests for principal investigators for the overall trial and each study site	____18_______
Access to data	29	Statement of who will have access to the final trial dataset, and disclosure of contractual agreements that limit such access for investigators	______16______
Ancillary and post-trial care	30	Provisions, if any, for ancillary and post-trial care, and for compensation to those who suffer harm from trial participation	____N/A_______
Dissemination policy	31a	Plans for investigators and sponsor to communicate trial results to participants, healthcare professionals, the public, and other relevant groups (eg, via publication, reporting in results databases, or other data sharing arrangements), including any publication restrictions	___17_______
	31b	Authorship eligibility guidelines and any intended use of professional writers	___19_____
	31c	Plans, if any, for granting public access to the full protocol, participant-level dataset, and statistical code	___18______

Appendices

For peer review only - http://bmjopen.bmj.com/site/about/guidelines.xhtml

Informed consent materials	32	Model consent form and other related documentation given to participants and authorised surrogates	__Supplementary file 2_______
Biological specimens	33	Plans for collection, laboratory evaluation, and storage of biological specimens for genetic or molecular analysis in the current trial and for future use in ancillary studies, if applicable	___N/A_____

*It is strongly recommended that this checklist be read in conjunction with the SPIRIT 2013 Explanation & Elaboration for important clarification on the items. Amendments to the protocol should be tracked and dated. The SPIRIT checklist is copyrighted by the SPIRIT Group under the Creative Commons "Attribution-NonCommercial-NoDerivs 3.0 Unported" license.

For peer review only
